# Disentangling Ethiopian Honey Bee (*Apis mellifera)* Populations Based on Standard Morphometric and Genetic Analyses

**DOI:** 10.3390/insects12030193

**Published:** 2021-02-25

**Authors:** Teweldemedhn Gebretinsae Hailu, Paul D'Alvise, Martin Hasselmann

**Affiliations:** 1 Department of Livestock Population Genomics, Institute of Animal Science, University of Hohenheim, Garbenstraße17, 70599 Stuttgart, Germany; paul.dalvise@uni-hohenheim.de (P.D.); martin.hasselmann@uni-hohenheim.de (M.H.); 2 Department of Animal Sciences, Aksum University, P.O. Box 314, Shire 1010, Ethiopia

**Keywords:** Ethiopia, Tigray, morphometrics, honey bee subspecies, classification, diversity

## Abstract

**Simple Summary:**

We conducted this population study of Ethiopian honey bees, using morphometric and genetic methods, to decipher their controversial classification. These honey bees are highly diverse and showed differentiation based on size and genetic information according to prevailing agro-ecological conditions, demonstrating morphological and molecular signatures of local adaptation. The results of both morphometric and genetic analyses suggest that Ethiopian honey bees differ from populations in the neighboring geographic regions and are characterized by extensive gene flow within the country, enhanced by honey bee colony trade. Consequently, future research that includes studying traits of vitality, behavior and colony performance of honey bees in remaining pocket areas of highland agro-ecological zones could contribute to the development of appropriate conservation management.

**Abstract:**

The diversity and local differentiation of honey bees are subjects of broad general interest. In particular, the classification of Ethiopian honey bees has been a subject of debate for decades. Here, we conducted an integrated analysis based on classical morphometrics and a putative nuclear marker (denoted r7-*frag*) for elevational adaptation to classify and characterize these honey bees. Therefore, 660 worker bees were collected out of 66 colonies from highland, midland and lowland agro-ecological zones (AEZs) and were analyzed in reference to populations from neighboring countries. Multivariate morphometric analyses show that our Ethiopian samples are separate from *Apis mellifera scutellata*, *A. m. jemenitica, A. m. litorea* and *A. m. monticola,* but are closely related to *A. m. simensis* reference. Linear discriminant analysis showed differentiation according to AEZs in the form of highland, midland and lowland ecotypes. Moreover, size was positively correlated with elevation. Similarly, our Ethiopian samples were differentiated from *A. m. monticola* and *A. m. scutellata* based on r7-*frag*. There was a low tendency towards genetic differentiation between the Ethiopian samples, likely impacted by increased gene flow. However, the differentiation slightly increased with increasing elevational differences, demonstrated by the highland bees that showed higher differentiation from the lowland bees (F_ST_ = 0.024) compared to the midland bees (F_ST_ = 0.015). An allelic length polymorphism was detected (denoted as *d*) within r7-*frag*, showing a patterned distribution strongly associated with AEZ (*X^2^* = 11.84, *p* < 0.01) and found predominantly in highland and midland bees of some pocket areas. In conclusion, the Ethiopian honey bees represented in this study are characterized by high gene flow that suppresses differentiation.

## 1. Introduction

The honey bee *Apis mellifera* belongs to a globally distributed species with a broad habitat range. This species has evolved to be thermally homeostatic, which has enabled its wide distribution throughout ecosystems, accompanied by diversification into distinct subspecies and ecotypes that differ morphologically and behaviorally [1], as well as genetically [2,3,4,5,6]. Several studies have been performed in order to elucidate their genetic divergence as well as their morphological plasticity, supporting the view of at least five major lineages denoted as C, M, A, O, Y of honey bees worldwide [3,7,8]. At the level of local subpopulation differentiation, honey bee traits such as high polyandry [9,10] enhance its fitness and productivity by increasing intracolonial genetic variability while reducing intercolonial differentiation. In addition, honey bee migratory and swarming behavior can contribute to an increased admixture of genetic material when occurring at higher rates, as described for African honey bees, for example [3,11,12].

Studies on the taxonomy, distribution and interrelationships among populations of the honey bee are not consistent in regions from which the species is hypothesized to have originated. In particular, there have been controversies in the classification, distribution and characterization of honey bee populations in northeast Africa and the Middle East among morphometric and molecular studies [1,2,3,7,13,14]. Ethiopian honey bees have been discriminated from neighboring populations and designated as a unique lineage and subspecies after decades of inconsistencies [3,15,16]. Former studies reported several subspecies in the country, including *A. m. jemenitica, A. m. scutellate* and *A. m. monticola* [17,18,19]. *A. m. jemenitica* is distributed in the semi-arid, arid and desert plains of northeast Africa and the Middle East, whereas *A. m. scutellata* inhabited the Savannah areas of eastern and southern Africa, and the corresponding patchy mountain forests are home for the darker and larger *A. m. monticola* [1,20].

A phenotype is the summation of genetic and environmental variances and their interactions [21], which can be deposed using relevant markers. For example, the mountain honey bee population in east Africa could be separated from neighboring lowland bees using classical morphometrics [22], as well as nuclear [6] and mitochondrial [23] genetic information. By conducting a whole-genome analysis on mountain forest (*A. m. monticola*) and savannah (*A. m. scutellata*) honey bees of Kenya, Wallberg et al. (2017) [6] identified an intriguing genomic region (denoted as r7) within the gene octopamine receptor beta-2R (LOC412896) located near a potential chromosomal breakpoint, showing striking variability between *A. m. monticola* and *A. m. scutellata*. Octopamine stabilizes hypoxia [24] and hypothermia [25] in locusts. In honey bees, it modulates responses to sucrose [26] and learning odors [27], visuals [28] and nursing [29] behaviors and the thermoregulatory fanning response [30]. Both physiological and behavioral traits are important parameters that could be associated with the local adaptation of honey bees. As an additional factor, the elevation of habitats and the accompanying local climate (e.g., rainfall, temperature, oxygen partial pressure, UV radiation) and vegetation can have a large impact and leave potential signatures of adaptation in species [31].

Apicultural activities have been affecting various honey bee subspecies in different parts of the world. This includes the rapid and widespread emergence of Africanized bees in South America [32], the complete substitution of an indigenous *A. m. mellifera* by commercial subspecies in central and northern Europe [33], the elimination of population structure among Iberian honey bees due to migratory beekeeping [34], and the reduction of genetic diversity in European honey bees [35]. A similar practice of honey bee colony marketing tradition exits in northern Ethiopia [36], but the extent and impact on the honey bee population has not been studied.

Ethiopia has contrasting agro-ecological zones (AEZs), where more than six million colonies are traditionally managed [37]. Its location in northeastern Africa, where there are contacts [7] between African (A) [1], Middle Eastern (O) and Ethiopian (Y) [3] lineages, makes it particularly important in honey bee population studies. Hence, classification and distribution of honey bees in the Ethiopian plateau has been a hot topic of research interest [15,17,18]. These honey bees have been described as a unique evolutionary lineage [3] and a subspecies [15] that differs from the honey bee populations in the neighboring countries. This was supported by a recent study on forewing geometric morphometrics and mitochondrial DNA analyses [16]. Geometric morphometry based on forewing venation—inherited from both parental lines—can efficiently separate honey bee populations [38,39,40], whereas the mitochondrial genomic region commonly known as COI-COII varies among honey bee lineages in sequence length and the frequency of characteristic P and Q motifs, which can provide sufficient information to elucidate evolutionary history of maternal lineages [41]. Hailu et al. (2020) [16] provided insights into evolutionary lineages, subspecies and mitochondrial haplotypes of Ethiopian honey bees based on AEZs and geographic location using forewing geometric morphometry and COI-COII.

Including genetic analyses based on nuclear markers associated with elevational adaptation in *A. mellifera* can help to better understand the distribution of honey bees in Ethiopia. In particular, molecular analysis using the recently identified nuclear marker r-7 [6] could disentangle signatures of genetic adaptation to various AEZs among the honey bees. Further, an in-depth analysis using integrated methods based on nuclear and mitochondrial DNA markers, together with classical morphometrics, would provide comprehensive insights into the population dynamics. In addition, classical morphometric analysis using the same sample could help to validate its conformity with previous reports of different methods that have focused on wing geometric morphometrics and COI-COII only. Establishing a solid morphological reference would support genetic studies and enhance honey bee research, conservation and production in the region. The null hypothesis states that honey bees inhabiting different AEZs of Ethiopia stand as a morphologically and genetically differentiated subspecies and belong to the populations in the neighboring countries. The alternative hypothesis would be that Ethiopian honey bees may have evolved as a unique subspecies due to agro-ecological isolation from populations in the neighboring countries, but are subject to extensive natural gene flow within Ethiopia, enhanced by anthropogenic activities.

Therefore, this study was conducted using selected classical morphometric traits [1] and a nuclear DNA fragment located in the aforementioned region r7 as a putative candidate for elevational adaptation in honey bees (r7-*frag*). We have analyzed eleven morphometric characteristics of size and the forewing that have enabled us to evaluate the selected morphological traits for separating Ethiopian honey bees in reference to neighboring subspecies and between AEZs within the country, and in association with environmental factors (elevation, location, temperature and rainfall). The integration of r7-*frag* sequence information provided insights into the local differentiation of Ethiopian honey bees among AEZs.

## 2. Materials and Methods

Samples of 660 worker honey bees out of 66 managed colonies were collected from nine sites in Tigray regional state (north) and two sites in the Wendogenet local area (south), representing highland, midland and lowland AEZs of Ethiopia (Table S1). Details on sampling and study sites are provided in [16]. The samples were imported to the University of Hohenheim (Germany) in compliance with formalities stipulated by the Ethiopian Institute of Biodiversity (reference code of permit letter: EBE71/160076/2018) and subjected to morphometric and genetic analyses.

### 2.1. Morphometric Analysis 

Based on their discriminatory power [1], the following morphometric characteristics were analyzed: metatarsus length, metatarsus width, metatarsal index, tibia, femur, total length of hind leg, forewing length, forewing width, distance of cubital vein "a", distance of cubital vein "b" and cubital index a/b (Figure S1). The right forewing and hind leg of each sample was detached, digitized using ZEISS Axiocam coupled with a ZEISS Stemi 305 microscope and processed with ZEN lite 2.1 software. A micrometer scale was integrated at the same magnification for calibration and size determination. The selected morphometric traits were measured out of the images of the forewings and hind legs using IC measure software. Data on the selected morphometric characteristics of individual bees were summarized and colony means were used in statistical analysis. Reference data of the same morphometric characteristics of colonies representing *A. m. scutellata* (19), *A. m. jemenitica* (18), *A. m. monticola* (9), *A. m. simensis* (15) and *A. m. litorea* (11) were obtained from Oberursel Bee Research Institute (Oberursel, Germany). The Kaiser–Meyer–Olkin (KMO) test was run as a measure of sampling adequacy (MSA) using JMP® Pro 15 software (SAS, Cary, NC, USA) and repeated until the KMO value reached a meritorious level (≥0.8) by removing morphometric variables that are less desirable for the analysis. Afterwards, principal component analysis (PCA) was run to calculate principal components, of which scores of the first two were used to form a scatter plot for detecting patterns of clustering among this study’s samples and in comparison to the reference subspecies. Next, a discriminant analysis was performed to confirm the established groups and determine distances between subspecies and ecotypes (highland, midland, lowland) within this study.

### 2.2. Molecular Analyses

Aliquots out of total DNA samples that were extracted for a preceding study [16] were used for PCR amplification of a nuclear fragment (r7-*frag*) located in the genomic region (581,079 to 583,086 within LOC412896, octopamine receptor beta 2R in chromosome 7), denoted as r7 [6]. PCR reactions using Dream Taq polymerase (Thermo Fisher, USA) and primers designed for this purpose (NCOI_Ex3in_Ex4_1fw: 5’-GTACCCATGGTTTTCTTCTCCCCCTTCTTTTC-3’, including the *NcoI* restriction enzyme site; PstI_Ex3inEx4in_1rev: 5’-CAGTCTGCAGTTCCACTATAACCGCTTTTCC-3’, including the *PstI* restriction enzyme site) were performed in a volume of 25 μL in duplicate, which were pooled to a volume of 50 μL, under the following conditions: initial denaturation at 94 °C for 120 s; followed by 33 cycles of denaturation at 94 °C for 20 s, 58 °C annealing for 30 s, and an elongation at 72 °C for 100 s; and final elongation at 72 °C for 4 minutes. PCR amplicons were observed by means of agarose gel electrophoresis (1.3%). DNA was purified using the MiniElute PCR purification kit (QIAGEN, Aarhus, Denmark). Afterwards, endonuclease restriction enzyme-mediated cloning was conducted using pGEM vector and *Escherichia coli* strain MJ109 (Promega, Madison, Wisconsin, USA). The pGEM vector and insert DNA were digested using *NcoI* and *PstI* endonuclease restriction enzymes by incubating at 37 °C for 90 minutes and at 80 °C for 20 minutes. Digested DNA was purified by ethanol precipitation, ligated with T4 Ligase by incubating overnight at 4 °C and used to transform competent *E. coli* cells. Transformants were plated on LB/ampicillin/IPTGX-Gal agar and incubated at 37 °C for 24 h. For each bee sample, eight white *E. coli* colonies were PCR-amplified using Dream Taq polymerase and T7 and SP6 primers to identify colonies with inserts of correct size. Colony PCR reactions were performed in a volume of 25 μL under the following conditions: initial denaturation at 94 °C for 120 s; followed by 33 cycles of denaturation at 94 °C for 20 s, 50 °C annealing for 20 s, and an elongation at 72 °C for 60 s; and final elongation at 72 °C for 3 minutes. Two clones that were estimated (by length) to contain alternative alleles of the r7-*frag* in each sample bee were selected based on agarose gel images and were further processed for plasmid isolation. Plasmids were isolated using Miniprep I (PeqGOLD/VWR Peqlab; Darmstadt, Germany), and aliquots of DNA premixed with T7 and SP6 primers were sent for Sanger sequencing (Microsynth, Balgach, Switzerland). A dataset of 94 r7-*frag* sequences was generated out of 62 sample bees from this study. In addition, 45 sequences out of 24 previously-collected honey bee samples from forest and savannah areas in Mount Kenya and the Mau region of Kenya [22] were generated as described above and included in the analysis (Table S2). PCR amplification of r7-*frag* failed for samples from Mount Kenya Forest, given by a putative chromosomal inversion (Wallberg et al., 2017, Supplementary Figure S8) [6].

Sequence data analysis was conducted using CLC Main Workbench 7.6.4 (QIAGEN, Aarhus Denmark) and DnaSP 6.12.03 software [42]. Subpopulations were defined based on the local area (Mugulat, Werie, Koyetsa, Wendogenet, Mau, Mount Kenya), agro-ecological zone (highland, midland, lowland) or habitat (mountain forest, savanna), geographic location (north, south), and country (Ethiopia, Kenya) of origin of the samples. Statistical analysis was run on population genetic diversity, divergence and gene flow using various parameters and models implemented in DnaSP: Genetic diversity was described in terms of the number of segregating sites (S), the average number of nucleotide differences (k) [43] and the average number of pairwise differences (π) [44], and the Watterson estimator (θw) [45] and Tajima’s D test [46] were conducted. The level of genetic differentiation was estimated using F_ST_ [47] and Kst* [48] and gene flow was estimated using Nm for a haplodiploid model organism (autosome, X-chromosome) based on Hudson et al. (1992) [49]. We ran the differentiation and gene flow analyses twice, excluding sites with sequence alignment gaps on the one hand and including a gap as a fifth state on the other hand, and presented the results accordingly. Mitochondrial COI-COII sequences obtained from the identical Ethiopian honey bee samples in a previous study [16] were included to compare levels of diversity and differentiation with that of r7-*frag*. To visualize the relationships between the populations, F_ST_ values were exported from DnaSP and used for tree construction using the neighbor-joining method [44] implemented in MEGA X [50].

Maximum likelihood analysis was conducted using 94 nucleotide sequences of honey bees from the highland, midland and lowland AEZs in four local areas of Ethiopia in order to elucidate their evolutionary relationships. First, the nucleotide substitution model that best fit to the dataset was identified using the model test option implemented in MEGA X [50]. Next, evolutionary history was inferred by the maximum likelihood algorithm and the Tamura–Nei model of nucleotide substitutions [51]. Initial trees for the heuristic search were obtained automatically by applying neighbor-joining and BioNJ algorithms to a matrix of pairwise distances estimated using the maximum composite likelihood (MCL) approach, and then selecting the topology with superior log likelihood value. A discrete Gamma distribution was used to model evolutionary rate differences among sites (5 categories (+G, parameter = 0.4080)). The rate variation model allowed for some sites to be evolutionarily invariable (+I, 37.83% sites). Two separate trees were generated: (a) all positions containing gaps and missing data were eliminated and there were a total of 1644 positions in the final dataset; (b) using all sites, with a total of 1996 positions in the final dataset.

### 2.3. Statistical Analyses

The distribution of ecotypes within Ethiopia was assessed using a logistic model and contingency analysis based on elevation, longitude, latitude, AEZ, annual precipitation, temperature, sampling site and local area as factors. In order to characterize the subspecies and ecotypes, mean values of the morphometric traits were summarized for the defined populations (Table 1). Pearson correlations were calculated among morphometric and genetic characters, as well as environmental factors (altitude, latitude, longitude) to assess associations with habitats of origin. Morphometric traits and environmental factors that showed significant correlations were further analyzed using a linear regression model to verify the effect of environmental factors. Normal distribution of the data was tested using goodness of fit.

The distribution of an allelic length polymorphism characterized by a sequence gap of 55 bp at position 858 to 915, denoted as *d* within r7-*frag*, was assessed using contingency analysis to determine its association with AEZs and the local areas. Furthermore, latent class analysis was performed by integrating the morphometric and molecular analyses using three nominal variables. The first variable, which is generated out of the classical morphometric analysis, deals with the highland, midland and lowland ecotypes. The second variable accounts for five mitochondrial haplotypes (Y1, Y2, Y3, A1, O5’) previously identified among this study’s samples. The third variable is a cluster with three levels generated out of r7-*frag* diversity parameters (number of segregating sites, average number of nucleotide differences, average number of pairwise differences, Watterson estimator) by defining populations according to sampling sites (Table S2). Considering the number of variables, as well as the smallest Bayesian information criterion (BIC) and Akaike information criterion (AIC) in accordance with Schreiber (2017) [52], the best fitting model was selected, clusters were saved and plotted on a graph to see possible distribution patterns. Statistical analysis was performed using JMP® Pro 15 software (SAS, Cary, NC, USA).

## 3. Results

### 3.1. Morphometric Analyses

Data obtained from the morphometric measurements were subjected to a series of complementary statistical analyses in order to determine clusters, classify subspecies and ecotypes and characterize them.

**Figure 1:**
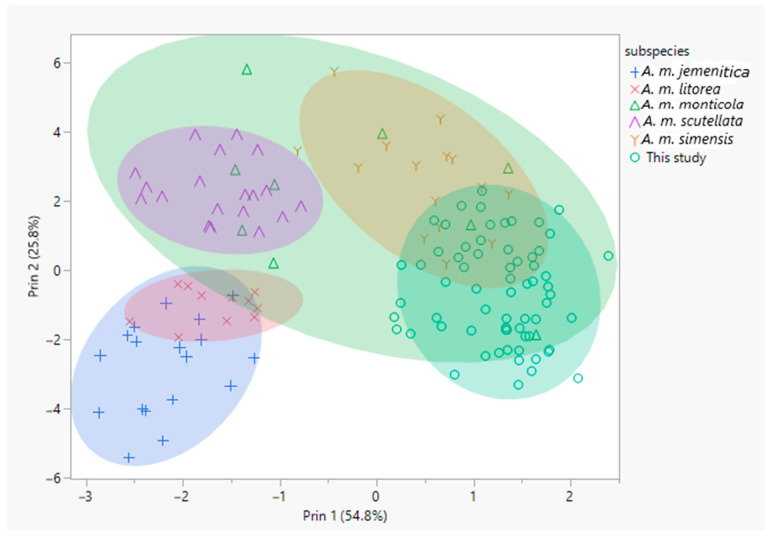
Scatter plot of principal component scores of this study’s samples and reference subspecies of *A. m. scutellata, A. m. jemenitica, A. m. simensis, A. m. litorea* and *A. m. monticola*. Scores were generated from nine morphometric characteristics: forewing length, forewing width, femur, tibia, metatarsal length, metatarsal width, total length of hind leg, cubital vein distance a and cubital vein distance b.

First, morphometric characteristics that were less desirable for the analysis were excluded by conducting the MSA test using KMO. It was possible to achieve an MSA value of 0.869 by removing cubital and metatarsal indices. The removal of the variables complies with the recommendations of Ruttner (1988) [1], who stated that indices and calculated values are problematic and should be excluded from morphometric multivariate classification of honey bee races. Next, principal component analysis was conducted based on correlations using the remaining nine morphometric characteristics and a scatter plot from the first two factor scores was used to identify patterns of distribution within the samples of this study and in comparison to the reference subspecies. Accordingly, the reference samples were separated into their respective groups. The samples of this study overlapped with the *A. m. simensis* reference group, and were separate from the other references (Figure 1).

Further, the separation of this study’s samples and the reference subspecies was verified by means of discriminant analysis that differentiated 92% (R^2^ = 0.84) of the samples into their respective groups. All colonies of the *A. m. simensis* reference were correctly assigned, demonstrating adequacy of the selected morphometric characteristics to separate Ethiopian honey bees. The discriminant analysis was repeated by labeling samples of this study as *A. m. simensis.* In this case, the model classified 89.8% (R^2^ = 0.73) of the samples into their respective groups. Based on the average squared distance, *A. m. simensis* was the closest to this study’s samples, followed by *A. m. monticola* (Table S3).

**Figure 2:**
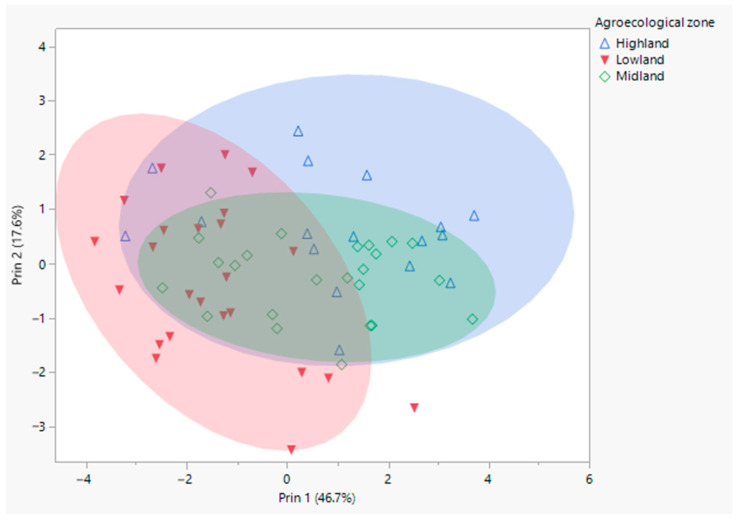
Scatter plot of principal component scores of this study’s samples assigned to their agro-ecological zone (highland, midland and lowland). Scores were generated from nine morphometric characteristics: forewing length, forewing width, femur, tibia, metatarsal length, metatarsal width, total length of hind leg, cubital vein distance a and cubital vein distance b.

Second, we applied the same procedures of principal component analysis as previously by excluding the reference samples in order to assess possible differentiation of groups within this study’s samples. A scatter plot with the scores of the first two principal components showed a slightly patterned distribution based on AEZs (Figure 2). Using discriminant analysis, 76% of the samples could be separated according to their agro-ecological zones of origin, as highland (72%), midland (70%) and lowland (83%). A considerable number of samples from the midland AEZ, a transitional AEZ between the highlands and lowlands, were grouped with the highland (16.7%; squared distance = 10.6) and lowland (12.5%; squared distance = 13.6) samples (Table S4). A logistic fitted model and contingency analysis showed that the distribution of these ecotypes differed significantly with elevation (*X*^2^ = 23.88; *p* < 0.01), AEZ (*X*^2^ = 51.3; *p* < 0.01), annual precipitation (*X*^2^ = 10.75; *p* < 0.01), temperature (*X^2^* = 13.38; *p* < 0.01), longitude (*X*^2^ = 9.25; *p* < 0.01), latitude (*X*^2^ = 24; *p* < 0.01), local area (*X^2^* = 14.97; *p* < 0.01) and sample site (*X^2^* = 78.14; *p* < 0.01).

Moreover, the morphometric traits were summarized in order to characterize the subspecies and ecotypes (Table 1). The mean values of the selected morphometric characters of the present samples were 8.29 mm forewing length, 3.02 mm forewing width and 7.27 mm hind leg length. Considering the mean of these morphometric characteristics separately, the closest subspecies to the sample of this study was *A. m. simensis* (cubital vein distance b), *A. m. monticola* (forewing width), *A. m. jemenitica* (cubital vein distance a, metatarsal width and tibia length) and *A. m. litorea* (forewing length, total length of hind leg, metatarsal length and femur). *A. m. monticola* had the longest forewing among the subspecies included in this study. This was followed by *A. m. simensis* and *A. m. scutellata*. Based on length of hind leg, *A. m. scutellata* and *A. m. simensis* were the first and second largest, respectively (Table 1). Comparing within this study’s samples based on AEZs, the highland samples were the largest, whereas the lowland samples were the smallest in size for all measured traits (Table 1). Consequently, there was a strong association of these traits with elevation. Specifically, forewing length was strongly correlated with elevation, whereas forewing, tibia and metatarsus width were moderately correlated with elevation. In addition, latitude showed a moderate inverse correlation with hind leg length and a moderate positive correlation with forewing length. Similarly, linear regression indicated that forewing length depends on elevation (F = 58.1; *p* < 0.01), latitude (F = 9.8; *p* < 0.01) and annual precipitation (F = 4.3; *p* < 0.05). Among the traits analyzed in this study, femur and tibia, metatarsus and tibia, forewing length and forewing width, as well as forewing and metatarsus length, are highly correlated with each other (Table S5).

### 3.2. Genetic Analyses

Wallberg et al. (2017) [6] identified a variable genomic region, denoted as r7, which depicted striking differentiation between mountain forest and savannah honey bees in Kenya, resulting from inversions that may govern local adaptation. This region is located on chromosome 7, including the gene octopamine receptor beta-2R (LOC412896) near a potential chromosomal breakpoint. We conducted genetic analysis within Ethiopian honey bees in different AEZs in comparison to neighboring Kenyan mountain forest and savannah bees using r7-*frag,* spanning 2 kb between position 581,079 to 583,086 within LOC412896.

Initially, we analyzed the nucleotide diversity of samples of this study. With a total number of 391 polymorphic sites and an average number of 30.44 nucleotide differences in r7-*frag*, the Ethiopian honey bee samples were found to have high nucleotide diversity (π = 0.0192; θw = 0.0486) in contrast to the diversity in mitochondrial COI-COII (Table 2).

When comparing the samples based on AEZs of origin, diversity consistently decreased with increasing elevation both in the Ethiopian and Kenyan samples. Hence, the highest numbers of segregating sites and nucleotide diversity were in the lowland AEZ, and the highland AEZ had the lowest numbers based on r7-*frag*, whereas the COI-COII did not show any pattern in these regards (Table 3).

All Tajimas’s D values were not significant. Considering the diversity among the three AEZs within each local area, the midlands were found to be the most diverse, followed by the lowlands of respective areas. On a local area basis, Koyetsa—located in northwestern zone of Tigray region where habitats of lineages O and Y overlap [16]—showed the highest diversity in both the nuclear and mitochondrial markers (r7-frag: θw = 0.0313; COI-COII: θw = 0.004). On the other hand, the Mugulat local area in the Eastern zone, which is the main source of honey bee colonies for different areas in the region, was the least polymorphic based on r7-*frag* (Table 2). The pattern of r7-*frag* diversity (number of segregating sites, average number of nucleotide differences, average number of pairwise differences, Watterson estimator) for the subpopulations defined based on sampling sites (Table 2) was verified by an intermediate level of inverse correlation (Pearson: −0.4) with increasing elevations, a small size of negative correlation with increasing precipitation, as well as medium level of direct association with temperature (Table S5). The effect of elevation was further tested using linear regression on Watterson estimator (F = 22.03; *p* < 0.01). Overall negative values of Tajima’s D test statistics indicated a skewed allele frequency distribution towards an excess of low-frequency mutations, commonly found after population expansion or a recent selective sweep, but also found in admixed populations. Further, similar negative D values were found between each elevational level, indicating no clear sign of local adaptation (Table 2 and Table 3).

Subsequently, the level of differentiation within this study and in comparison to Kenyan mountain forest (*A. m. monticola*) and savannah (*A. m. scutellata*) reference samples was assessed using F_ST_ and Kst*. We verified that *A. m. monticola* and *A. m. scutellata*, included here as references, can be separated using r7-*frag* at first. There was a significant difference between the honey bees of Kenyan mountain and savannah areas when analyzing r7-*frag*, both by excluding sites with sequence alignment gaps (F_ST_ = 0.0798; Kst* = 0.015) and considering the gap as a fifth state (F_ST_ = 0.08487; Kst* = 0.017). The Kenyan samples followed a consistent differentiation according to their habitats. That is, the honey bees from the savanna area of the Mau region differentiated more significantly from the corresponding mountain forest bees (F_ST_ = 0.0766; Kst* = 0.025) compared to a geographically distant population in the savanna area of Mount Kenya (F_ST_ = 0.055; Kst* = 0.015), (Figure S4A1). This was more pronounced when sites with sequence alignment gaps were considered in the analysis (Figure S4A2). Second, we compared the honey bee samples from Ethiopia and Kenya. The Ethiopian samples of this study significantly differed from the Kenyan references of both *A. m. monticola* and *A. m. scutellata* (*p* < 0.001), (Figure S4C1, C2). On the basis of AEZ, the *A. m. monticola* reference was more differentiated from the highland (F_ST_ = 0.1093) and lowland (F_ST_ = 0.101) compared to the midland (F_ST_ = 0.0468) AEZs of Ethiopian honey bees in this study, mainly when all sites were considered in the analysis (Table 4; Figure S4B).

Next, we measured and compared the levels of differentiation within this study’s samples of Ethiopian honey bees using r7-*frag* and COI-COII. Based r7-*frag,* the samples could be significantly separated between local areas as well as between AEZs within each local area, which consistently increased with increasing elevation (Table 5a). In contrast, COI-COII could not sufficiently differentiate the samples based on AEZs or local areas (Table 5b). Despite the statistical significance, the differentiation between AEZs among the samples of this study was generally small (Table 4 and Table 5; Figures S4A,B and S5).

The level of population structuring was validated using a gene flow estimate (Nm) [49]. The honey bees from northern Ethiopia demonstrated a high level of gene flow between local areas as well as AEZs. For example, there was an abundant flow between highland and midland in Koyetsa (Nm = 36.78) and Mugulat (Nm = 17.87) areas (Table S6). On the other hand, there was relatively low level of flow between the highlands and lowlands of the Werie area (Nm = 2.23), which is in line with the high level of differentiation discussed previously.

When comparing the overall rates of flow pooled on the basis of AEZs, the midland—a transitional AEZ—exchanged abundantly with the highlands (Nm = 14.19) and lowlands (Nm = 12.22). Gene flow between the local areas seems to be less related with geographic distance, as demonstrated by the highest value (Nm = 17.27) being between Koyetsa and Werie, and the lowest (Nm = 6.21) being between Mugulat and Werie areas. In contrast, the samples from southern Ethiopia showed a low level of flow between AEZs, similar to that of the Kenyan reference samples (Table S7).

### 3.3. Integrated Analysis Using Morphometrics and Molecular Data

The morphometric ecotypes, mitochondrial haplotypes [16] and genetic diversity based on r7-*frag* of this study’s samples were combined in a latent class analysis (Table S8), aiming to obtain an integrated view of Ethiopian honey bee differentiation along elevational levels. Considering the number of variables as well as the smallest Bayesian information criterion (BIC) and Akaike information criterion (AIC), a three cluster model was run in accordance with Schreiber (2017) [52]. Three of the variables showed significant effects in the model (*p* < 0.01). Therefore, the three clusters were saved in a separate column and plotted in a graph to see the patterns of distribution of the clusters.

**Figure 3:**
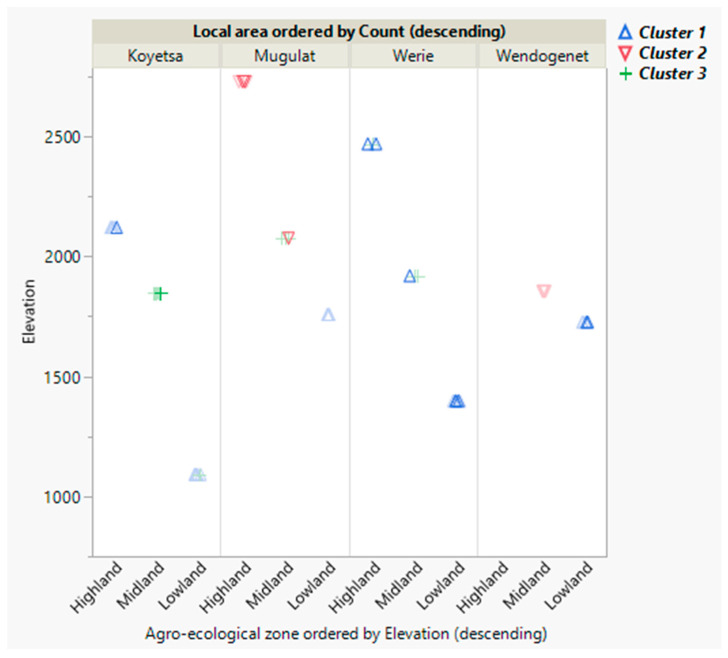
Scatter plot of most likely clusters of Ethiopian honey bee samples of this study based on latent class analysis using classical morphometric ecotypes (highland, midland, lowland), mitochondrial haplotypes of this study (Y1, Y2, Y3, A1, O5’) as previously reported [16] and three clusters based on diversity parameters (number of segregating sites, average number of nucleotide differences, average number of pairwise differences, Watterson estimator) using a nuclear marker on chromosome seven, denoted as r7.4. Discussion.

The results showed that cluster 1 was distributed in all areas, cluster 2 was found in Mugulat (north) and Wendogenet (south), whereas cluster 3 occupied the midland AEZ of all local areas in the north (Koyetsa, Mugulat, Werie). The samples from southern Ethiopia (Wendogenet) were fully separated between the lowland and midland AEZs, whereas the samples from the north were rather mixed between the AEZs, in particular in the midlands. On the other hand, the highland area of Mugulat, which is the highest mountain peak in this study, represented a population clearly separated from that of the corresponding midland and lowland areas (cluster 2), although the reverse did not hold, indicating a unidirectional flow of the honey bees in this area, down the AEZs. In the Werie area the flow of colonies seemed to be upward (cluster 1 and 3), whereas there was no sign of admixture in the lowland AEZs (Figure 3).

## 4. Discussion

### 4.1. Morphometric Analyses

The results of this classical morphometric analysis indicate that the Ethiopian honey bees represented in this study are differentiated from *A. m. jemenitica, A. m. scutellata, A. m. monticola* and *A. m. litorea* (Figure 1, Table S3). This study’s samples formed one main group that overlapped with *A. m. simensis,* which is in agreement with a previous classical morphometric study [15]. Similarly, a more recent study based on forewing geometric morphometrics [16] grouped 88% of this samples with *A. m. simensis*, whereas the rest were sporadically distributed between other subspecies. Formerly, there had been debates on the number of honey bee subspecies in Ethiopia, in which Radloff and Hepburn (1997) [17] noted *A. m. jemenitica A. m. bandasii* and *A. m. scutellata*, whereas Amssalu et al. (2004) [18] reported *A. m. jemenitica, A. m. scutellata* and *A. m. monticola*, among two others. Contrarily, Radloff and Hepburn (2000) [53] described Ethiopian honey bees as a subgroup of *A.* *m. scutellata*, differentiated from populations in eastern and southern Africa. Later, Meixner et al. (2011) [15] designated Ethiopian honey bees as a distinct subspecies, *A. m. simensis*, based on morphometric analysis within Ethiopia and in comparison to references of neighboring subspecies. However, a recent study on these samples [16] showed the presence of maternal lineage O in northern Ethiopia. This indicates that methodological variations in honey bee population studies may lead to contrasting results.

By analyzing this study’s samples separately, we observed a tendency towards morphometric clustering between AEZs, whereby the samples were grouped based on their habitats of origin, in the form of highland, midland and lowland ecotypes (Figure 2, Table S4). Differentiation between the highland and lowland ecotypes was larger compared to the midland, which demonstrated intermediate squared distances and morphometric mean values. The highland ecotypes were characterized as the largest, whereas the lowland ecotypes were the smallest in size (Table 1). Therefore, forewing length and width, metatarsus width and tibia positively correlated with increasing elevation (Table S5). In line with this, there was a strong association between elevation and centroid size of the forewing based on geometric morphometrics in a previous study using the same samples [16]. Similarly, a linear relationship between size and elevation was reported in honey bees of Ethiopia [15] and Kenya [22]. Generally, mountain honey bees are larger than their neighbors at lower elevations [20]. According to Abou-Shaara (2015) [54], forewing size plays a key role in the thermal adaptation of honey bees. Hence, populations within a subspecies that occupy a broad range could show morphological plasticity [1,22]. Ethiopia is composed of diverse agro-ecological zones, including dry mountains in the central and northern highlands, transitional savannah midlands, and peripheral desert lowlands separating the country from neighboring populations in the east, south and west. The highlands are the coldest, the lowlands are the hottest, whereas the midlands are transitional between the two AEZs (Table S1). Consequently, the vegetation cover, growth period and flowering patterns vary between AEZs, which could influence the distribution of the honey bees. Thus, most of the highland (72%) and lowland (83%) samples were differentiated according to their habitats of origin, although a considerable proportion of the midland samples overlapped with both AZEs. This most likely explains the significantly varied distribution of these ecotypes with elevation, temperature and annual precipitation.

It is important to note that morphometric mean values, particularly the forewing size of *A. m. simensis* and the samples of this study (Table 1), appeared to be markedly different, which could be related to differences in the elevational distribution of samples (2292 and 1931 masl, respectively) sampling season and year, and operating person and equipment. However, we obtained a scanned copy of the reference *A. m. simensis* samples that were collected in 1998, along with an image of a measuring scale. This study’s samples were directly collected in 2018, scanned and measured.

### 4.2. Genetic Analyses

The nucleotide diversity of r7-*frag* varied between agro-ecological zones as well as local areas and showed in general substantially higher levels of diversity when compared to COI-COII sequence data (Table 2 and Table 3). Given the maternal inheritance of the non-recombining mitochondrial CO-COII markers, this finding is conclusive. A previously conducted phylogenetic analysis using the maximum likelihood method based on the mitochondrial COI-COII marker showed that some (17.7%) of the Ethiopian honey bees, mainly samples from Koyetsa area, clustered with lineage O, indicating an overlap between the lineages O and Y in this area. Koyetsa hosted the highest number (four out of the five) of mitochondrial haplotypes, whereas the lowest number was found in Mugulat [16]. Koyetsa is the sampling site with the lowest elevation and northernmost location in Ethiopia. In contrast, the local area Mugulat includes a mountain peak, which is the highest elevation among all the sampling sites in this study. Highland honey bees in East Africa are larger, gentler and darker [1,20], and hence could be discriminated from neighboring lowland populations using classical morphometric [22], mitochondrial [23] and nuclear [6] genetic analyses.

Interestingly, the diversity of r7-*frag* was inversely related with increasing elevation, which might be indicative of a rather reduced effective population size at higher elevations. Based on the negative values of Tajima’s D, one could assume the fixation of a favorable allele (selective sweep). We found the marker r7-*frag* to be characterized by a significant allelic length polymorphism among Ethiopian honey bees (Figure S4; Table 4), which could support this assumption. However, negative Tajima’s D values and the underlying excess of low frequency mutations can also be a result after a population size expansion or population admixture. As we noticed a reasonably high level of gene flow among the sample areas, we cannot fully disentangle these alternatives at the current stage and more genome-wide data will be needed.

Ethiopian samples of this study were differentiated more from *A. m. monticola* compared to *A. m. scutellata* reference subspecies, in contrast to classical morphometric analysis (Table S3). Moreover, *A. m. monticola* was more diverged from the Ethiopian highland compared to the midland honey bees, considering the allelic length polymorphism. Particularly, a large segment of sequence gap *d* was observed in a large proportion of the samples from the Ethiopian highlands, as opposed to the trend in Kenya (Table S9). Wallberg et al. (2017) [6] hypothesized the genomic region of r7 to be a candidate for the local adaptation of East African honey bees to highland environments. Within this study’s samples, an increased differentiation was detected with increasing elevational differences when using r7-*frag* sequence information (Table 5). Interestingly the allelic length polymorphism (*d*) was associated with the respective environments. In this regard, a considerable proportion of the highland and midland bees were characterized by *d*, unlike the lowland bees from Koyetsa and Wendogenet areas, where no *d* was found. This was most abundant in the highland AEZ of Werie area (71%), where the most pronounced level of divergence between the highland and lowland honey bees was detected (Figure S5; Table 5). Inversely, the least divergence (F_ST_ = 0.06; Table 5) was found in the Koyetsa area, since only 33% of the highland bees displayed *d*, whereas there was none in the lowland of this area (Table S9); implying an upward direction of the high magnitude gene flow (Table S6) in the honey bees. Although the level of differentiation within this study was generally small, as demonstrated by low F_ST_ values (Table 4 and Table S7), we were able to differentiate the honey bees of Ethiopia between local areas as well as between AEZs within each area (Table 5). Using the same genetic marker r7-*frag*, a striking divergence was identified between the mountain forest and savannah populations of unmanaged honey bees in neighboring Kenya, which was also observed in this study among the Kenyan reference samples [6]. In contrast, the populations in northern Ethiopia seemed to be partly mixed-up due to directional colony transportation; which gave rise to inter-area and inter-AEZ variation in genetic diversity with a low level of differentiation. Beekeepers in the colder highland areas of eastern zone of Tigray such as Mugulat are specialized in colony multiplication. Swarms are considered an important source of income and have little chance of escaping the beekeepers [55]. Colonies originating from these highland areas are traded across AEZs in the region [36]. This is reflected in the unidirectional gene flow down the mountains that we observed in our analyses (Table S9, Figure 3). Conversely, the flow of colonies in Koyetsa and Werie areas seems to be in upward direction, as the highland bees’ signature *d* was non-existent in the former and negligible in the latter. In line with this, a preliminary honey bee colony market survey [56] indicated that suppliers in the Werieleke district collect feral colonies from the river valley of Werie, whereas in the Ganta-Afeshum district, such as the Mugulat area, they rear their own colonies. The lowlands of Werie and Koyetsa areas are characterized as extensive rangelands with higher vegetation cover, where trapping of swarms is likely in contrast to the highland areas of Werie and Mugulat [36]. Hence, the source colonies for the market are unmanaged feral honey bees in this area. According to Harpur et al. (2012) [57], management increases genetic diversity in the honey bee by promoting admixture, although this statement sparked arguments for [58] and against [59]. Migratory beekeeping was reported to have eliminated previously existing population structure in Iberian honey bees [34] and the introduction of commercial breeds replaced local populations in Central Europe [33]. A more recent study indicated that a loss of genetic diversity and a reduction in the level of admixture have occurred in European honey bees during the last century [35]. Population genetic diversity—which influences adaptation and tolerance to stress in honey bees [33]—can be affected by anthropogenic activities, including breeding and habitat management [35], as well as selection and recombination [60]. High polyandry enhances fitness and productivity by increasing intracolonial genetic variability in honey bees [9,10].

## 5. Conclusions

This study showed that the honey bee subspecies of Ethiopia can be separated using as few as nine selected classical morphometric traits, and they largely overlap with *A. m. simensis*. Across three elevational levels, clear morphometric differentiation seems to be impacted by potential phenotypic plastic responses to varying environmental conditions. Our genetic analyses indicate rather high levels of gene flow between the local sites, likely driven by anthropogenic activities such as colony trading. We obtained additional insights into the local honey bee populations from different AEZs and identified a nuclear genetic fragment (r7-*frag*) containing an allelic length polymorphism (*d*) found to be associated with elevation. This association, despite the presence of gene flow, might be indicative of a locally adapted allelic constellation that needs to be further investigated. In addition, future research should include studies on honey bee vitality, behavior and colony performance, in order to provide more robust arguments, supporting conservation and management efforts to protect local populations.

## Figures and Tables

**Table 1:** Mean values (mm) of morphometric characteristics of size in this study’s samples and the reference subspecies obtained from the Oberursel Bee Research Institute (Germany). The lower table shows morphometric means of highland, lowland and midland ecotypes of this study and the statistical significance of agro-ecological zone (AEZ) within the Ethiopian samples.

Subspecies	Femur	Tibia	Metatarsus Length	Metatarsus Width	Length of Hind Leg	Forewing Length	Forewing Width	Cubital Vein Distance ^a^	Cubital Vein Distance ^b^	Cubital Index
*A. m. jemenitica*	2.36	2.92	1.82	1.04	7.11	8.13	2.80	0.42	0.19	2.26
*A. m. litorea*	2.42	2.99	1.85	1.05	7.26	8.37	2.90	0.44	0.19	2.33
*A. m. monticola*	2.46	3.07	1.95	1.09	7.49	8.83	3.01	0.49	0.22	2.23
*A. m. scutellata*	2.53	3.13	1.95	1.10	7.60	8.65	2.98	0.46	0.20	2.38
*A. m. simensis*	2.51	3.08	1.98	1.08	7.57	8.70	3.05	0.51	0.23	2.22
This study	2.40	2.93	1.90	1.02	7.27	8.29	3.02	0.51	0.23	2.24
Highland	2.41 ^ab^	2.96 ^a^	1.91 ^a^	1.05 ^a^	7.29 ^a^	8.43 ^a^	3.05 ^a^	0.51 ^a^	0.24 ^a^	2.20 ^a^
Lowland	2.38 ^b^	2.88 ^b^	1.88 ^a^	1.00 ^b^	7.26 ^a^	8.16 ^c^	2.98 ^b^	0.51 ^a^	0.23 ^a^	2.19 ^a^
Midland	2.41 ^a^	2.95 ^a^	1.91 ^a^	1.02 ^c^	7.28 ^a^	8.31 ^b^	3.03 ^a^	0.52 ^a^	0.23 ^a^	2.30 ^a^
Significance (AEZ)	*	**	*	**		**	**			

Note: Levels not connected by same letter are significantly different for the specified character in a column; * *p* < 0.05, ** *p* < 0.01.

**Table 2:** Population genetic parameters for the highland, midland and lowland agro-ecological zones (AEZs) of four local areas of Ethiopia and Kenyan reference samples from mountain forest and savannah areas of Mau region and Mount Kenya. For Ethiopian samples, data are given for r7-*frag* (first rows in each group) and COI-COII (second rows in each group) and for Kenyan samples, r7-frag.

Country	Geographic Region	Local Area	AEZ	Number of Segregating Sites (S)	Nucleotide Diversity			Tajima’s D
					Average Number of Differences (k)	Average Number of Pairwise Differences (π)	Watterson Estimator (θw)	
Ethiopia	North	Mugulat	Highland	66	24.75	0.0156	0.0172	−0.179
				0.0	0.0	0.0	0.001	
			Midland	111	33.47	0.0211	0.0277	−1.014
				0.0	0.0	0.0	0.0	
			Lowland	79	28.62	0.0180	0.0212	−0.663
				5	1.6	0.004	0.005	
			Total Mugulat	173	29.98	0.0189	0.0296	−1.543
				5	0.56	0.001	0.002	
		Werie	Highland	75	27.10	0.0171	0.0207	−0.762
				1	0.4	0.001	0.001	
			Midland	73	29.40	0.0185	0.0223	−0.697
				1	0.6	0.001	0.001	
			Lowland	95	29.20	0.0184	0.0220	−0.486
				0.0	0.0	0.0	0.0	
			Total Werie	169	30.41	0.0192	0.0299	−1.294
				2	0.36	0.001	0.001	
		Koyetsa	Highland	82	28.31	0.0178	0.0212	−0.486
				6	3.06	0.006	0.006	
			Midland	106	31.03	0.0196	0.0252	−0.861
				5	2.67	0.006	0.005	
			Lowland	92	29.44	0.0186	0.0232	−0.585
				4	2.13	0.005	0.004	
			Total Koyetsa	181	30.21	0.0190	0.0313	−1.392
				7	2.4	0.005	0.004	
		Total North		354	30.65	0.0193	0.0460	−2.045
				8	1.61	0.004	0.004	
	South	Wendogenet	Midland	64	25.62	0.0161	0.0196	−0.095
				2	0.8	0.002	0.002	
			Lowland	92	29.31	0.0185	0.0225	−0.368
				0.0	0.0	0.0	0.0	
			Total South	122	29.20	0.0184	0.0248	−0.752
				2	0.4	0.001	0.002	
	Total Ethiopia			391	30.44	0.0192	0.0486	−2.09
				10	1.5	0.003	0.005	
		Mau	MF	69	23.89	0.0151	0.0161	−0.128
			MS	124	28.81	0.0182	0.0243	−0.841
Kenya		Mount Kenya	MKS	118	30.71	0.0194	0.0230	−0.515
			Savanna	183	30.68	0.0196	0.0287	−1.111
		Total Kenya		207	29.91	0.0189	0.0302	−1.349

MF: mountain forest in Mau region; MS: savanna land in Mau region; MKS: savanna land in Mount Kenya; savanna: samples from the savanna of both Mount Kenya and Mau region. Note: rows indicated as total refer to the data obtained from the analysis of all samples for each region. All Tajimas’s D values are not significant.

**Table 3:** Population genetic parameters for highland, midland and lowland agro-ecological zones (AEZs) pooled from four local areas of Ethiopia and Kenyan reference samples from two local areas (Mau and Mount Kenya). For Ethiopian samples, data are given for r7-*frag* (first rows in each group) and COI-COII (second rows in each group) and for Kenyan samples, r7-*frag*.

Country	AEZ	Number of Segregating Sites (S)	Nucleotide Diversity			Tajima’s D
			Average Number of Nucleotide Differences (k)	Average Number of Pairwise Difference (π)	Watterson Estimator (θw)	
Ethiopia	Highland	142	28.10	0.0177	0.0258	−1.099
		6	1.57	0.003	0.004	
	Midland	166	30.48	0.0192	0.0308	−1.432
		7	1.43	0.003	0.004	
	Lowland	234	30.91	0.0195	0.0364	−1.418
		5	1.5	0.003	0.003	
Kenya	Mountain forest	69	23.89	0.0151	0.0161	−0.128
	Savanna	183	30.68	0.0196	0.0287	−1.111

**Table 4:** Genetic differentiation based on r7-*frag*: distance, F_ST_ (lower triangle) and statistical significance, Kst* including permutation test (upper triangle) between honey bees from highland, midland and lowland agro-ecological zones (AEZs) of Ethiopia, pooled on the basis of AEZs as well as mountain forest and savannah areas of Kenyan reference samples from mount Kenya and the Mau region. Analysis was performed by excluding sites with sequence alignment gaps (first rows) and considering gaps as a fifth state (second rows) separately.

	This Study	All Highlands	All Midlands	All Lowlands	MF	MS	MKS	All Savannah
This study					0.005 ***	0.005 ***	0.007 ***	0.006 ***
					0.006 **	0.009 ***	0.006 **	0.007 ***
All highlands			0.004 *	0.006 **	0.018 ***	0.013 ***	0.015 ***	0.009 ***
			0.005 *	0.012 ***	0.023 ***	0.013 **	0.015 **	0.008 *
All midlands		0.0149		0.005 **	0.011 **	0.010 **	0.012 ***	0.008 ***
		0.0215		0.007 ***	0.012 **	0.013 ***	0.011 **	0.009 ***
All lowlands		0.0236	0.0153		0.012 ***	0.010 ***	0.012 ***	0.008 ***
		0.0797	0.0297		0.017 ***	0.025 ***	0.015 ***	0.017 ***
MF	0.0542	0.0775	0.0463	0.062		0.025 ***	0.026 ***	0.015 ***
	0.0697	0.1093	0.0468	0.101		0.031 **	0.029 **	0.017 **
MS	0.0332	0.0356	0.0415	0.039	0.0766		0.015 ***	
	0.0411	0.0487	0.04	0.0732	0.1014		0.021^**^	
MKS	0.0496	0.057	0.0545	0.0547	0.0947	0.055		
	0.0874	0.0413	0.0671	0.1647	0.1205	0.0923		
	0.02767	0.03223	0.03196	0.03324	0.0798			
All savannah	0.03957	0.02033	0.03206	0.09598	0.08487			

Note: superscripts indicate statistical significance of permutation test: *: 0.01 < *p* < 0.05; **: 0.001< *p* <0.01; ***: *p* < 0.001; MS: mountain forest in Mau region; MS: savanna land in Mau region; MKS: savanna land in Mount Kenya; All savannah: samples from the savannah areas of both Mau region and mount Kenya; all highlands: samples from the highland AEZs of Mugulat, Werie and Koyetsa; All midlands: samples from the midland AEZs of Mugulat, Werie, Koyetsa and Wendogenet; all lowlands: samples from the highland AEZs of Mugulat, Werie, Koyetsa and Wendogenet; This study: samples of this study collected from different local areas in Ethiopia.

**Table 5:** Genetic differentiation: distance, F_ST_ (lower triangle) and statistical significance, Kst* (upper triangle) between honey bees from highland, midland and lowland agro-ecological zones (AEZs) of Mugulat, Werie, Koyetsa and Wendogenet areas of Ethiopia, as well as mountain forest (*A.* *m. monticola*) and savannah (*A. m. scutellata*) reference samples in Mount Kenya and Mau region of Kenya. Analysis was performed by excluding sites with sequence alignment gaps (first rows) and considering gaps as a fifth state (second rows) separately.

**(** **a) Analysis based on a nuclear marker on chromosome seven, denoted as r7-frag, using Ethiopian and Kenyan honey bee samples.**															
**Local Area**	**AEZ**	**Mugulat**			**Werie**			**Koyetsa**			**Wendogenet**		**Mau**		**Mount Kenya**
		**Highland**	**Midland**	**Lowland**	**Highland**	**Midland**	**Lowland**	**Highland**	**Midland**	**Lowland**	**Midland**	**Lowland**	**MF**	**MS**	**MKS**
Mugulat	Highland		0.007 ^ns^	0.022 *	0.044 ***	0.012 ^ns^	0.031 **	0.031 **	0.015 *	0.027 ***	0.042 *	0.024 **	0.024 *	0.019 **	0.022 **
			0.005 ^ns^	0.026 *	0.052 *	0.012 ^ns^	0.033 *	0.020 *	0.012 ^ns^	0.035 **	0.041 *	0.029 **	0.035 *	0.018 *	0.019 **
	Midland	0.02		0.009 ^ns^	0.029 **	−0.006 ^ns^	0.021 *	0.006 ^ns^	−0.001 ^ns^	0.011 **	0.031 *	0.009 ^ns^	0.016 *	0.012 *	0.009 *
		−0.01		0.008 ^ns^	0.041 *	0.008 ^ns^	0.020 *	0.004 ^ns^	0.002 ^ns^	0.019 ***	0.027 *	0.013 *	0.022 *	0.012 *	0.006 ^ns^
	Lowland	0.07	0.04		0.039 *	0.011 ^ns^	0.033 **	0.018 **	0.012 *	0.018 *	0.047 *	0.018 *	0.026 *	0.013 *	0.014 *
		0.081	0.013		0.062 **	0.010 ^ns^	0.033 **	0.015 ^ns^	0.013 ^ns^	0.026 **	0.043 *	0.022 *	0.033 *	0.023 **	0.013 *
Werie	Highland	0.094	0.081	0.076		0.011 ^ns^	0.035 **	0.018 *	0.023 **	0.021 *	0.053 *	0.029 **	0.054 ***	0.025 ***	0.023 **
		0.164	0.180	0.240		0.028 ^ns^	0.057 **	0.034 *	0.041 **	0.062 **	0.062 *	0.064 **	0.074 **	0.030 **	0.039 ***
	Midland	0.013	−0.013	0.025	0.024		0.020 *	0.010 ^ns^	0.0004 ^ns^	0.008 ^ns^	0.035 ^ns^	0.006 ^ns^	0.023 *	0.012 *	0.012 *
		0.008	−0.037	0.013	0.133		0.016 ^ns^	0.003 ^ns^	0.002 ^ns^	0.010 ^ns^	0.025 ^ns^	0.006 ^ns^	0.022 ^ns^	0.014 *	0.011 ^ns^
	Lowland	0.091	0.069	0.118	0.120	0.078		0.020 *	0.019 **	0.019 *	0.035 *	0.021 **	0.038 ***	0.026 ***	0.028 ***
		0.109	0.055	0.110	0.219	0.028		0.020 *	0.021 *	0.022 *	0.037 **	0.026 **	0.043 ***	0.033 **	0.029 **
Koyetsa	Highland	0.070	0.012	0.061	0.041	0.060	0.10		0.001 ^ns^	0.006 ^ns^	0.034 *	0.008 ^ns^	0.031 ***	0.012 **	0.009 ^ns^
		0.066	−0.001	0.042	0.095	0.001	0.05		0.0001 ^ns^	0.011 ^ns^	0.021 ^ns^	0.015 ^ns^	0.029 *	0.015 *	0.011 ^ns^
	Midland	0.037	−0.022	0.042	0.076	−0.005	0.07	0.01		0.011 *	0.027 *	0.009 ^ns^	0.018 *	0.010 *	0.013 *
		0.025	−0.031	0.039	0.173	−0.022	0.06	−0.01		0.017 **	0.024 *	0.013 *	0.017 *	0.011 *	0.011 *
	Lowland	0.108	0.039	0.064	0.039	0.026	0.07	0.03	0.04		0.031 *	0.010 ^ns^	0.029 **	0.017 ***	0.015 **
		0.190	0.107	0.108	0.272	0.021	0.08	0.06	0.10		0.034 *	0.009 ^ns^	0.037 **	0.036 ***	0.023 **
Wendogenet	Midland	0.016	0.059	0.113	0.091	0.040	0.06	0.09	0.05	0.07		0.034 *	0.054 **	0.029 **	0.030 **
		0.103	0.088	0.118	0.175	0.033	0.07	0.04	0.05	0.09		0.036 **	0.048 **	0.028 **	0.031 **
	Lowland	0.074	0.023	0.060	0.083	0.005	0.08	0.03	0.02	0.04	0.09		0.032 **	0.016 **	0.015 **
		0.142	0.057	0.060	0.285	−0.002	0.07	0.05	0.06	0.01	0.09		0.036 **	0.035 ***	0.020 **
Mau	MF	0.061	0.032	0.068	0.142	0.063	0.10	0.10	0.03	0.09	0.11	0.10		0.025 ***	0.026 ***
		0.097	0.051	0.080	0.277	0.039	0.13	0.07	0.02	0.16	0.11	0.14		0.031 **	0.029 ***
	MS	0.057	0.037	0.057	0.087	0.056	0.088	0.035	0.023	0.067	0.092	0.050	0.077		0.015 **
		0.064	0.066	0.137	0.121	0.098	0.148	0.053	0.035	0.243	0.109	0.212	0.121		0.021 **
Mount Kenya	MKS	0.103	0.021	0.054	0.110	0.070	0.123	0.030	0.046	0.076	0.124	0.055	0.095	0.055	
		0.077	−0.003	0.039	0.187	0.047	0.106	0.006	0.028	0.139	0.124	0.094	0.101	0.092	
**(b) Analysis based on the mitochondrial COI-COII region using Ethiopian honey bee samples.**															
**Local Area**	**AEZ**	**Mugulat**				**Werie**				**Koyetsa**				**Wendogenet**	
		**Highland**		**Midland**	**Lowland**	**Highland**	**Midland**	**Lowland**		**Highland**	**Midland**	**Lowland**		**Midland**	**Lowland**
Mugulat	Highland			nc	0.025 ^ns^	0.057 ^ns^	0.214 ^ns^	nc		0.233 ^ns^	0.298 ^ns^	0.450 ^ns^		0.014 ^ns^	nc
				0.103 *	0.004 ^ns^	0.066 ^ns^	0.080	0.138 *		0.096	0.101 ^ns^	0.178 ^ns^		0.023 ^ns^	0.031 ^ns^
	Midland	0.0000			0.025 ^ns^	0.057 ^ns^	0.214 ^ns^	nc		0.233 ^ns^	0.298 ^ns^	0.450 ^ns^		0.087 ^ns^	nc
		0.1857			0.085 ^ns^	0.075 ^ns^	0.243 **	0.106 ^ns^		0.194 *	0.190 *	0.296 *		0.200 **	0.126 *
	Lowland	0.0000		0.0000		−0.033 ^ns^	−0.008 ^ns^	0.025 ^ns^		0.020 ^ns^	0.054 ^ns^	0.145 ^ns^		−0.028 ^ns^	−0.024 ^ns^
		nc		0.063		0.086 *	0.096 *	0.147 *		0.031 ^ns^	0.042 ^ns^	0.094 ^ns^		0.033 ^ns^	0.006 ^ns^
Werie	Highland	0.0000		0.0000	0.0000		0.069 ^ns^	0.057 ^ns^		0.116 ^ns^	0.191 ^ns^	0.329 ^ns^		−0.012 ^ns^	0.000 ^ns^
		0.1858		0.0615	0.0970		0.115 *	0.161 *		0.099 ^ns^	0.104 ^ns^	0.203 *		0.131 **	0.042 ^ns^
	Midland	0.2500		0.2500	nc	0.1667		0.214 ^ns^		0.096 ^ns^	0.124 ^ns^	0.268 ^ns^		0.043 ^ns^	0.156 ^ns^
		0.1901		0.3232	0.0719	0.1667		0.066 ^ns^		0.102 ^ns^	0.097 ^ns^	0.192 *		0.042 ^ns^	0.047 ^ns^
	Lowland	0.0000		0.0000	0.0000	0.0000	0.2500			0.233 ^ns^	0.298 ^ns^	0.450 ^ns^		0.087 ^ns^	nc
		0.3333		0.4316	0.2111	0.2521	0.1122			0.155 **	0.148 *	0.245 *		0.065 ^ns^	0.105 ^ns^
Koyetsa	Highland	0.3429		0.3429	0.0533	0.2973	0.2143	0.3429			−0.068 ^ns^	−0.049 ^ns^		0.105 ^ns^	0.180 ^ns^
		0.2686		0.3558	0.1110	0.2553	0.2552	0.3119			−0.060 ^ns^	−0.061 ^ns^		0.106 ^ns^	0.057 ^ns^
	Midland	0.4286		0.4286	0.1136	0.3947	0.2576	0.4286		nc		−0.071 ^ns^		0.155 ^ns^	0.245 ^ns^
		0.2704		0.3503	0.1211	0.2685	0.2533	0.3082		nc		−0.048 ^ns^		0.113 ^ns^	0.071 ^ns^
	Lowland	0.6000		0.6000	0.2723	0.5581	0.4605	0.6000		nc	nc			0.278 ^ns^	0.401 ^ns^
		0.4276		0.5225	0.2606	0.4399	0.4223	0.4743		nc	nc			0.201 ^ns^	0.153 ^ns^
Wendogenet	Midland	0.0000		0.0000	0.0000	0.0000	0.1250	0.0000		0.2927	0.3659	0.5217			0.030 ^ns^
		0.0698		0.3099	0	0.2473	0.0741	0.2376		0.3219	0.3268	0.4842			
	Lowland	0.0000		0.0000	0.0000	0.0000	0.2500	0.0000		0.3429	0.4286	0.6000		0.0000	
		0.0614		0.0843	nc	0.0160	0.0250	0.1680		0.2160	0.2361	0.3959		nc	

Superscripts indicate statistical significance of permutation test: ^ns^ = not significant; *: 0.01 < *p* < 0.05; **: 0.001 < *p* < 0.01; ***: *p* < 0.001. Note: MS: mountain forest in Mau region; MS: savanna land in Mau region; MKS: savanna land in Mount Kenya; nc = not calculated. Similarly, a phylogenetic tree analysis using the maximum likelihood method showed that the Ethiopian honey bees of this study are more admixed and showed a low tendency of clustering (Figure S2). However, a pairwise comparison revealed that there was a slightly increasing differentiation with increasing elevational difference. Accordingly, the level of differentiation of the highlands from the lowlands was higher (F_ST_ = 0.0236) compared to that of midland AEZs (F_ST_ = 0.0149). An increased level of differentiation between the highland and lowland honey bees was observed (F_ST_ = 0.0797; *p* < 0.001) when all sites were considered in the analysis, indicating a substantial contribution of allelic length polymorphism between the samples of the distinct AEZs (Figure S4; Table 4). In this regard, a large segment of allelic length polymorphism was detected at position 858 to 915 of r7-*frag* (Figure S3). Here, considerable proportions of the samples from the highland (45.8%) and midland (25%) AEZs exhibited a gap of 55 bp (hereafter denoted as *d*), whereas none of the lowland bees from Koyetsa and Wendogenet areas showed *d*. The proportion of the samples characterized by *d* was more pronounced (71%) among the highland bees in the Werie local area. Therefore, divergence of the highland from lowland honey bees was more significant in this area (F_ST_ = 0.219) than others, whereas the least was observed in Koyetsa (F_ST_ = 0.06), (Figure 2; Table 5), where only 33% of the highland samples were characterized by *d* (Figure S3). Overall, the distribution of *d* was strongly associated with AEZs (*X^2^* = 11.84, *p* < 0.01).

## Data Availability

The sequence datasets generated out of this study were deposited into GenBank (accession numbers: MW228891 to MW229029) and the morphometric data are available on request from the corresponding author.

## Supplementary Materials

The following are available online at https://www.mdpi.com/2075-4450/12/3/193/s1, Figure S1: Selected morphometric traits out of forewing (A) and hind leg (B) of the sample honey bee analyzed in this study: femur, tibia, metatarsus length and width, metatarsal index, hind leg length, forewing length and width, cubital vein distances a and b and cubital index. Figure S2: Phylogenetic trees of this study’s sequences out of three agro-ecological zones (AEZs) in four local areas. The evolutionary history was inferred by using the maximum likelihood method and the Tamura–Nei model [51]. Initial trees for the heuristic search were obtained automatically by applying neighbor-joining and BioNJ algorithms to a matrix of pairwise distances estimated using the maximum composite likelihood (MCL) approach, and then selecting the topology with a superior log likelihood value. A discrete Gamma distribution was used to model evolutionary rate differences among sites (5 categories (+G, parameter = 0.4080)). The rate variation model allowed for some sites to be evolutionarily invariable (+I, 37.83% sites). This analysis involved 94 nucleotide sequences. (A) All positions containing gaps and missing data were eliminated and there were a total of 1644 positions in the final dataset. (B) All sites used, with a total of 1996 positions in the final dataset. Evolutionary analyses were conducted in MEGAX [50]. Figure S3: Demonstration and analysis of an allelic length polymorphism, containing a sequence gap of 55 nucleotides, denoted as *d*. (A) A partial view of this study’s sample sequences indicated with IDs on the left margin showing position 858 to 915 of the putative candidate r7-frag on chromosome seven in the honey bee. (B) Contingency analysis of *d* distribution by agro-ecological zones (AEZs), which were strongly associated (X2 = 11.84, *p* < 0.01). (C) Contingency analysis of *d* distribution in three local areas (Mugulat, Werie, Koyetsa) of northern Ethiopia, consisting of highland, midland, lowland and the Wendogenet area in southern Ethiopia, consisting of midland and lowland AEZs. Figure S4: Neighbor-Joining trees based on pairwise distances (F_ST_) using r7-frag sequence information obtained from honey bees from different agro-ecological zones (AEZs) in Mugulat, Werie, Koyetsa and Wendogenet areas of Ethiopia and *A. m. monticola* and *A. m. scutellata* samples from Kenya. Samples per AEZ (highland, midland, lowland) were pooled. (1) F_ST_ analysis conducted by excluding sites with sequence alignment gaps. (2) F_ST_ analysis conducted by including gaps as a fifth state, amplifying distances. (A) Differentiation between honey bee populations from highland, midland and lowland AEZs in reference to samples from Mau forest area (MF) and savannah area of Mount Kenya (MKS) and the Mau region (MS), depicting the divergence of Ethiopian highland bees away from Kenyan mountain forest bees. (B) Differentiation between honey bee populations from highland, midland and lowland AEZs in reference to *A. m. monticola* and *A. m. scutellata samples* from Kenya, indicating that *A. m. scutellata* is relatively closer to the honey bees in all AEZs of Ethiopia. (C) Divergence of this study’s samples from Ethiopia relative to Kenyan reference *A. m. monticola* and *A. m. scutellata*. B and C indicate a marked divergence of *A. m. monticola* from both *A. m. scutellata* and the Ethiopian samples of this study. Evolutionary analysis was performed using MEGA X [50] and genetic differentiation was performed with DnaSP 6.12.03 software [42]. Note: all highlands: samples from the highland AEZs of Mugulat, Werie and Koyetsa; all midlands: samples from the midland AEZs of Mugulat, Werie, Koyetsa and Wendogenet; all lowlands: samples from the highland AEZs of Mugulat, Werie, Koyetsa and Wendogenet; this study: samples of this study collected from Ethiopia. Figure S5. Neighbor-joining (NJ) trees based on pairwise distance (F_ST_) using r7-frag sequence information obtained from honey bees in highland, midland, lowland agro-ecological zones (AEZs) in Mugulat, Werie, Koyetsa and Wendogenet areas of Ethiopia and *A. m. monticola* and *A. m. scutellata* samples from Kenya. (A) NJ tree based on F_ST_ values analyzed by excluding sites with sequence alignment gaps. (B) NJ tree based on F_ST_ values analyzed by including gaps as a fifth state, showing marked differentiation between honey bees of lowland and highland AEZs in Werie due to a large segment of allelic length polymorphism, mainly at position 858 to 915 of r7-frag, where a sequence gap of 55 bp (denoted as *d*) characterized the highland in contrast to the lowland bees in these areas. Evolutionary analysis was performed using MEGA X [50], whereas genetic differentiation was conducted in DnaSP 6.12.03 software [42]. Table S1: Description of the sample colonies used in this study (number sampled and codes given), sample site location (administrative zone, latitude, longitude), elevation, agro-ecological zone (AEZ), temperature and precipitation, Table S2: Number of sequences generated out of this study’s samples from highland, midland and lowland agro-ecological zones (AEZs) in four local areas (Mugulat, Werie, Koyetsa, Wendogenet) of Ethiopia and Kenyan reference samples from mountain forest and savannah areas of Mau region and mount Kenya. The sequences generated in this study refer to a genomic fragment denoted as r7-frag on chromosomes seven within the gene LOC412896, octopamine receptor beta-2R, Table S3: Subspecies classification and squared distances of this study’s samples to reference honey bee subspecies of *A. m. jemenitica*, *A. m. litorea*, *A. m. monticola*, *A. m. scutellata* and *A. m. simensis*, obtained from the Oberursel Bee Research Institute (Germany), Table S4: Classification of this study’s samples as highland, lowland and midland ecotypes and corresponding distances between each sample and the groups based on classical morphometric characteristics (femur, tibia, metatarsus length and width, metatarsal index, hind leg length, forewing length and width, cubital vein distances a and b, cubital index), Table S5: Correlations (lower triangle) between morphometric characteristics (femur, tibia, metatarsus length and width, metatarsal index, hind leg length, forewing length and width, cubital vein distances a and b, cubital index), genetic diversity parameters (S, π, θw) defined on an agro-ecological zone and local area basis, and environmental factors (elevation, longitude, latitude), as well as corresponding probability values (upper triangle) of this study’s samples, Table S6: Gene flow (Nm) between highland, midland and lowland agro-ecological zones (AEZs) of three local areas in northern Ethiopia based on a nuclear marker on chromosome seven, denoted as r7-frag. Analysis was performed with the sequence format of diploid, X-Chromosome to deal with the haplodiploid nature of the honey bee using DnaSP 6.12.03 software by excluding sites with sequence alignment gaps (lower triangle) and considering gaps as a fifth state (upper triangle) separately [42], Table S7: Gene flow (Nm) among pooled populations by areas and agro-ecological zones (AEZs). Analysis was performed with the sequence format of diploid, X-Chromosome to deal with the haplodiploid nature of the honey bee using DnaSP 6.12.03 software by excluding sites with sequence alignment gaps (lower triangle) and considering gaps as a fifth state (upper triangle) separately [42], Table S8: Three variables used in latent class analysis: classical morphometrics, r7-frag nucleotide diversity cluster and COI-COII DraI haplotypes previously identified in this study;s samples [16], Table S9: Summary of samples with sequence gap (denoted as *d*) at position 858 to 915 of the nuclear marker r7-*frag* on chromosome seven in the honey bee.
