# Supplementary material for: Disentangling Ethiopian Honey Bee (Apis mellifera) Populations Based on Standard Morphometric and Genetic Analyses
Source: Insects. 2021 Feb 25;12(3):193. doi: 10.3390/insects12030193 (PMC7996220; doi:10.3390/insects12030193)
Supplement: Supplementary file 1 [file insects-12-00193-s001.pdf]

Table S1. Description of the sample colonies used in this study (number sampled and codes given), sample site location (administrative zone, latitude, longitude), elevation, agro-ecological zone (AEZ), temperature and precipitation.

Local Area Grouped as	Site	Administrative Zone	Number of Colonies Sampled (codes)	Latitude (°N)	Longitude (°E)	Elevation (masl)	Average Temperature (°C)*	Annual Precipitation (mm)*	AEZ
Mugulat[a]	Mugulat	Eastern	6(111, 112, 113, 114, 115, 116)	14.215	39.358	2730	15.28	467	highland
	Adikebero	Eastern	6(121, 122, 123, 124, 125, 126)	14.267	39.305	2078	17.81	461	midland
	Simret	Central	6(131, 132, 133, 134, 135, 136)	13.629	38.889	1757	20.8	620	lowland
Werie[a]	Kolageble	Central	6(211, 212, 213, 214, 215, 216)	14.141	39.068	2468	17.87	492	highland
	Tsedya	Central	6(221, 222, 223, 224, 225, 226)	14.053	39.192	1918	19.59	495	midland
	Werie bridge	Central	6(231, 232, 233, 234, 235, 236)	13.846	38.9978	1397	22.17	547	lowland
Koyetsa [a]	Shire	Northwest	6(311, 312, 313, 314, 315, 316)	14.179	38.333	2120	18.9	508	highland
	Adidaero	Northwest	6(321, 322, 323, 324, 325, 326)	14.316	38.165	1848	20.5	487	midland
	Aditsetser	Northwest	6(331, 332, 333, 334, 335, 336)	14.336	37.630	1090	25.39	461	lowland
Wendogenet [b]	Wendogenet	West Arsi	6(411, 412, 413, 414, 415, 416)	7.098	38.643	1856	17.34	963	midland
	Entaye	Sidama	6(421, 422, 423, 424, 425, 426)	7.026	38.608	1725	18.07	963	lowland

Note: N: north; E: East; masl: meters above sea level; AEZ: agroecological zone; mm: millimeter; °C: degree Celsius; Samples marked in bold are not sequenced; * Averages for the period 1961 to 1990; retrieved from Global Agroecological Zones; [a] Local areas from Tigray region in northern Ethiopia; [b] Local area from southern Ethiopia; Source: Hailu et al. (2020).

Table S2. Number of sequences generated out of this study samples from highland, midland and lowland agroecological zones (AEZs) in four local areas (Mugulat, Werie, Koyetsa, Wendogenet) of Ethiopia and Kenyan reference samples from mountain forest and savannah areas of Mau region and mount Kenya. The sequences generated in this study refers to a genomic fragment denoted as r7-*frag* on chromosomes seven within the gene LOC412896, Octopamine receptor beta-2R.

Country	Local Area	AEZ	Number of Sequences	Number of Worker Bees Sequenced
Ethiopia	Mugulat	Highland	8	5
		Midland	9	6
		Lowland	7	5
	Werie	Highland	7	6
		Midland	6	5
		Lowland	11	6
	Koyetsa	Highland	9	6
		Midland	11	6
		Lowland	9	6
	Wendogenet	Midland	7	6
		Lowland	10	5
			94	62
Kenya	Mau	Forest (MF)	10	6
		Savannah (MS)	17	10
	Mount Kenya	Savannah (MKS)	18	9
			45	25

Table S3. Subspecies classification and squared distances of this study samples to reference honey bee subspecies of *A. m. jemenitica, A. m. litorea, A. m. monticola, A. m. scutellata, A. m. simensis* obtained from Oberursel Bee Research Institute (Germany).

Sample ID	Classified as	Squared Distance				
		A. m. jemenitica	*A. m. litorea*	*A. m. monticola*	*A. m. scutellata*	*A. m. simensis*
111	*A. m. simensis*	75.44	59.75	36.94	49.04	20.92
112	*A. m. simensis*	78.19	52.28	33.72	44.35	21.62
113	*A. m. simensis*	68.98	52.42	41.65	53.27	21.71
114	*A. m. simensis*	60.36	47.48	26.04	43.09	10.65
115	*A. m. simensis*	61.02	50.46	39.67	55.24	33.53
116	*A. m. simensis*	42.74	31.98	21.87	32.76	14.17
121	*A. m. simensis*	63.80	53.25	38.23	48.00	21.37
122	*A. m. simensis*	67.61	54.13	37.01	53.39	15.78
123	*A. m. simensis*	61.74	48.90	39.30	46.26	18.92
124	*A. m. simensis*	46.96	34.53	22.67	31.61	8.26
125	*A. m. simensis*	64.12	45.54	28.12	37.81	10.68
126	*A. m. simensis*	72.55	54.67	43.13	49.18	17.38
131	*A. m. simensis*	101.51	86.66	73.30	82.68	39.66
132	*A. m. simensis*	66.53	53.54	36.06	54.65	18.97
133	*A. m. simensis*	55.17	47.05	38.32	51.22	23.16
134	*A. m. simensis*	60.42	49.53	39.84	53.08	21.04
135	*A. m. simensis*	61.31	53.92	43.75	59.33	25.02
136	*A. m. simensis*	58.20	47.34	46.02	55.40	28.11
211	*A. m. simensis*	57.35	37.90	27.07	32.43	11.51
212	*A. m. simensis*	49.30	34.90	26.15	31.66	10.31
213	*A. m. simensis*	57.84	36.78	17.36	27.87	4.44
214	*A. m. simensis*	64.59	42.25	26.66	35.30	9.65
215	*A. m. simensis*	65.85	46.11	37.05	42.51	15.92
216	*A. m. simensis*	61.13	41.57	32.26	36.32	14.28
221	*A. m. simensis*	44.53	34.12	29.57	38.46	16.96
222	*A. m. simensis*	47.35	33.74	38.61	37.83	21.94
223	*A. m. simensis*	34.03	20.31	16.52	19.53	7.62
224	*A. m. simensis*	57.96	42.72	39.97	48.46	20.84
225	*A. m. simensis*	80.45	59.64	46.16	58.69	25.99
226	*A. m. simensis*	44.86	24.90	20.28	22.68	8.44
231	*A. m. simensis*	51.50	39.14	32.51	45.29	19.65
232	*A. m. simensis*	45.55	36.43	25.66	43.19	18.38
233	*A. m. simensis*	54.03	41.45	36.48	45.59	19.11
234	*A. m. simensis*	44.74	35.48	32.89	45.01	21.56
235	*A. m. simensis*	47.13	40.98	37.89	47.42	21.49
236	*A. m. simensis*	42.82	29.20	28.98	36.53	19.33
311	*A. m. simensis*	54.90	41.26	34.40	49.71	23.04
312	*A. m. simensis*	51.77	38.99	31.92	38.07	17.82
313	*A. m. simensis*	53.50	45.82	34.33	50.54	19.09
314	*A. m. simensis*	41.70	31.47	39.17	44.10	27.72
315	*A. m. simensis*	67.19	50.91	28.49	46.20	10.33
316	*A. m. simensis*	72.51	56.78	37.96	56.79	18.66
321	*A. m. simensis*	47.47	35.37	28.28	39.73	17.53
322	*A. m. simensis*	49.97	36.23	32.28	44.38	25.05
323	*A. m. simensis*	64.76	46.12	38.53	48.48	21.85
324	*A. m. simensis*	65.97	49.71	44.18	54.59	24.65
325	*A. m. simensis*	63.28	47.69	35.20	47.38	15.64
326	*A. m. simensis*	34.39	23.69	34.02	30.07	21.56
331	*A. m. simensis*	54.84	41.73	35.27	43.79	15.66
332	*A. m. simensis*	46.00	38.02	38.97	46.29	24.50
333	*A. m. simensis*	69.05	54.00	52.10	56.73	29.69
334	*A. m. simensis*	53.74	44.82	41.19	47.67	20.03
335	*A. m. simensis*	80.22	67.87	66.38	78.53	39.94

336	*A. m. simensis*	34.06	28.01	33.09	32.54	18.61
411	*A. m. simensis*	53.49	45.65	34.16	42.33	16.54
412	*A. m. simensis*	98.10	79.08	62.67	74.66	31.01
413	*A. m. simensis*	83.68	61.25	59.42	62.18	33.49
414	*A. m. simensis*	111.03	86.57	74.71	79.18	36.33
415	*A. m. simensis*	88.07	71.99	59.36	66.81	29.14
416	*A. m. simensis*	64.73	51.08	52.79	58.34	30.87
421	*A. m. simensis*	40.61	38.40	52.33	45.73	32.49
422	*A. m. simensis*	71.57	62.96	60.24	69.55	33.15
423	*A. m. simensis*	80.75	69.97	73.59	77.20	42.69
424	*A. m. simensis*	104.86	90.51	78.40	89.42	61.32
425	*A. m. simensis*	96.22	84.55	74.79	87.00	59.23
426	*A. m. simensis*	102.89	87.57	86.21	90.16	65.59

Table S4. Classification of this study samples as highland, lowland and midland ecotypes and corresponding distances between each sample and the groups based on classical morphometric characters (femur, tibia, metatarsus length and width, metatarsal index, hind leg length, forewing length and width, cubital vein distances a and b, cubital index).

ID	Actual	Distance			Predicted
		Highland	Lowland	Midland	
111	Highland	16.68	24.08	16.66	Midland
112	Highland	22.47	43.43	30.95	Highland
113	Highland	10.09	13.46	10.24	Highland
114	Highland	7.96	12.14	8.31	Highland
115	Highland	15.34	26.21	20.01	Highland
116	Highland	4.48	11.36	8.57	Highland
211	Highland	5.32	17.43	7.38	Highland
212	Highland	9.11	11.03	6.5	Midland
213	Highland	4.99	20.39	10.18	Highland
214	Highland	4.49	16.85	6.03	Highland
215	Highland	4.46	12.32	4.46	Highland
216	Highland	6.4	16.93	8.63	Highland
311	Highland	8.22	7.42	9.11	Lowland
312	Highland	7.76	13.41	10.29	Highland
313	Highland	8.02	4.82	9.11	Lowland
314	Highland	10.86	5.97	8.46	Lowland
315	Highland	3.9	11.14	7.84	Highland
316	Highland	5.1	9.64	7.51	Highland
Average		8.65	15.45	10.57	
131	Lowland	30.1	21.07	29.39	Lowland
132	Lowland	9.2	8.03	10.93	Lowland
133	Lowland	14.61	7.17	11.82	Lowland
134	Lowland	9.19	3.63	7.16	Lowland
135	Lowland	13.62	5.87	13.87	Lowland
136	Lowland	16.03	7.08	13.38	Lowland
231	Lowland	3.42	4.86	3.56	Highland
232	Lowland	7.35	6.89	10.69	Lowland
233	Lowland	5.23	5.3	6.34	Highland
234	Lowland	12.47	4.68	10.08	Lowland
235	Lowland	9.26	4.05	8.27	Lowland
236	Lowland	6.05	8.7	7.36	Highland
331	Lowland	8.83	3.82	5.22	Lowland
332	Lowland	14.02	6.74	8.24	Lowland
333	Lowland	12.01	11.98	5.66	Midland
334	Lowland	10.71	3.08	5.32	Lowland
335	Lowland	18.02	7.25	12.51	Lowland
336	Lowland	13.44	7.28	10.01	Lowland
421	Lowland	29.97	17	22.47	Lowland
422	Lowland	17.81	4.97	10.73	Lowland

423	Lowland	27.03	12.14	19.48	Lowland
424	Lowland	30.44	22.93	30.27	Lowland
425	Lowland	35.2	20.33	32.04	Lowland
426	Lowland	36.24	22.24	31.43	Lowland
Average		16.26	9.46	13.59	
121	Midland	12.61	19.7	12.2	Midland
122	Midland	4.75	4.34	5.41	Lowland
123	Midland	6.96	10.47	6.72	Midland
124	Midland	1.85	7.04	3.27	Highland
125	Midland	5.08	15.26	4.97	Midland
126	Midland	9.06	8.84	5.87	Midland
221	Midland	7.52	6.59	8.47	Lowland
222	Midland	12.13	10.67	9.96	Midland
223	Midland	5.3	12	5.92	Highland
224	Midland	6.01	3.74	3.12	Midland
225	Midland	9.85	18.39	9.85	Midland
226	Midland	9.38	18.5	9.56	Highland
321	Midland	5.8	6.02	6.05	Highland
322	Midland	12.21	12.67	9.71	Midland
323	Midland	11.69	14.76	7.35	Midland
324	Midland	6.29	6.91	4.23	Midland
325	Midland	2.42	5.55	1.11	Midland
326	Midland	17.35	11.99	13.58	Lowland
411	Midland	11.28	13.33	10.18	Midland
412	Midland	12.71	13.56	7.68	Midland
413	Midland	16.59	19.48	9.32	Midland
414	Midland	17.42	21.65	13.36	Midland
415	Midland	14.47	15.11	11.19	Midland
416	Midland	11.61	6.82	5.17	Midland
Average		9.60	11.81	7.68	

Table S5. Correlations (lower triangle) between morphometric characters (femur, tibia, metatarsus length and width, metatarsal index, hind leg length, forewing length and width, cubital vein distances a and b, cubital index), genetic diversity parameters (S, π, θw) based on r7-*frag* defined on agroecological zone and local area basis, and environmental factors (Elevation, Longitude, Latitude), as well as corresponding probability values (upper triangle) of this study samples.

	Femur	Tibia	Metatarsus L	Metatarsus W	Hind leg L	Metatarsal index	Forewing L	Forewing W	Cubital vein a	Cubital vein b	Cubital index	S	K	π	θw	Lat	Long	Ele	Temp	Precip
Femur		***	***	***	***	ns	***	***	***	ns	**	**	ns	ns	ns	ns	**	***	***	ns
Tibia	0.76		***	***	***	ns	***	***	**	ns	ns	**	**	**	ns	ns	***	***	***	ns
Metatarsus L	0.58	0.72		***	***	***	***	***	ns	ns	ns	ns	ns	ns	ns	ns	**	***	***	ns
Metatarsus W	0.42	0.56	0.36			***	***	***	ns	ns	ns	ns	ns	ns	ns	***	***	***	***	***
Hind leg L	0.58	0.60	0.60	0.14		***	ns	***	**	ns	ns	ns	ns	ns	ns	***	ns	ns	***	***
Metatarsal index	–0.05	–0.02	–0.45	0.66	–0.33		***	ns	ns	ns	ns	ns	ns	ns	ns	***	ns	ns	ns	***
Forewing L	0.36	0.57	0.36	0.69	0.07	0.38		***	ns	ns	ns	**	ns	ns	ns	***	***	***	***	***
Forewing W	0.54	0.66	0.56	0.51	0.34	0.04	0.71		**	ns	ns	ns	ns	ns	ns	ns	***	***	***	ns
Cubital vein a	0.38	0.25	0.18	0.01	0.27	–0.13	0.01	0.27		***	***	ns	ns	ns	ns	ns	ns	ns	ns	ns
Cubital vein b	–0.10	0.03	0.07	–0.11	–0.06	–0.15	0.09	0.01	–0.43		***	ns	ns	ns	ns	ns	ns	ns	ns	ns
Cubital index	0.28	0.13	0.05	0.09	0.18	0.04	–0.03	0.14	0.80	–0.88		ns	ns	ns	ns	ns	ns	ns	ns	ns
S	–0.30	–0.27	–0.07	–0.23	–0.06	–0.20	–0.25	–0.24	–0.12	–0.08	–0.04		***	***	***	ns	**	***	***	**
K	–0.17	–0.25	–0.09	–0.16	–0.07	–0.13	–0.22	–0.21	–0.03	–0.17	0.07	0.90		***	***	**	ns	***	***	**
π	–0.17	–0.24	–0.09	–0.16	–0.07	–0.13	–0.22	–0.21	–0.03	–0.18	0.08	0.91	1.00		***	**	ns	***	***	**
θw	–0.10	–0.16	0.00	–0.16	–0.03	–0.20	–0.21	–0.13	0.05	–0.22	0.14	0.90	0.98	0.98			ns	***	***	ns
Lat	–0.22	–0.18	–0.20	0.32	–0.48	0.48	0.48	0.04	–0.18	–0.09	–0.04	0.24	0.27	0.27	0.20		ns	ns	**	***
Long	0.28	0.34	0.29	0.45	0.15	0.23	0.49	0.32	–0.08	0.14	–0.13	–0.26	–0.13	–0.13	–0.19	0.08		***	***	ns
Ele	0.32	0.50	0.42	0.53	0.20	0.21	0.66	0.52	0.00	0.11	–0.06	–0.41	–0.40	–0.40	–0.39	0.14	0.63		***	ns
Temp	-0.38	-0.56	-0.50	-0.36	-0.39	0.01	-0.41	-0.46	-0.05	-0.19	0.09	0.40	0.38	0.39	0.36	0.30	-0.66	-0.88		**
Precip	0.17	0.08	0.14	-0.36	0.42	-0.47	-0.51	-0.08	0.14	0.12	0.00	-0.25	-0.28	-0.28	-0.23	-0.98	-0.08	-0.18	-0.26	

*** = $p < 0.01$, ** = $p < 0.05$, ns = not significant, L: length, W: width, Lat: latitude, Long: longitude, Ele: elevation, Tem: temperature, Precip: precipitation; Note: Population genetic diversity parameters of segregating sites (S), average number of pairwise difference (π) and Watterson estimator (θw) were calculated by defining sample sequences of r7-*frag* within the gene LOC412896 based on their AEZ and local area of sample origin using DnaSP 6.12.03 software.

Table S6. Gene flow (Nm) between highland, midland and lowland agroecological zones (AEZs) of three local areas in northern Ethiopia based on a nuclear marker on chromosome seven denoted as r7-*frag*. Analysis was performed with the sequence format of diploid, X-Chromosome to deal with the haplo-diploid nature of the honey bee using DnaSP 6.12.03 software. by excluding sites with sequence alignment gaps (lower triangle) and considering gap as a fifth state (upper triangle) separately [35].

Local Area	AEZ	Mugulat			Werie			Koyetsa		
		Highland	Midland	Lowland	Highland	Midland	Lowland	Highland	Midland	Lowland
Mugulat	Highland		n/c	3.79	1.70	42.17	2.73	4.68	12.78	1.42
	Midland	17.87		25.92	1.52	n/c	5.71	n/c	n/c	2.79
	Lowland	4.88	9.75		1.06	25.67	2.69	7.64	8.29	2.75
Werie	Highland	1.76	2.59	2.14		2.18	1.19	3.18	1.60	0.89
	Midland	22.96	n/c	9.33	6.99		11.55	336.33	n/c	15.23
	Lowland	2.27	3.82	2.00	2.23	3.56		5.90	n/c	5.90
Koyetsa	Highland	3.46	15.64	4.27	7.35	7.13	3.69		n/c	5.50
	Midland	5.96	n/c	7.34	3.49	n/c	5.10	36.78		2.93
	Lowland	2.47	6.36	3.86	5.70	12.22	4.59	20.55	8.16	

n/c: not calculated.

Table S7. Gene flow (Nm) among pooled populations by areas and agroecological zones (AEZs). Analysis was performed with the sequence format of diploid, X-Chromosome to deal with the haplo-diploid nature of the honey bee using DnaSP 6.12.03 software by excluding sites with sequence alignment gaps (lower triangle) and considering gap as a fifth state (upper triangle) separately [35].

Local Areas in Northern Ethiopia				Wendogenet Area, Southern Ethiopia			Northern Ethiopian Samples Pooled Based on AEZs				Kenya			
	Mugulat	Werie	Koyetsa		Midland	Lowland		Highlands	Midlands	Lowlands		MF	MS	MKS
Mugulat		7.06	10.72				Highlands		10.26	4.08	MF		2.43	2.95
Werie	6.21		15.87	Midland		3.19	Midlands	14.19		9.95	MS	3.16		3.28
Koyetsa	10.95	17.27		Lowland	3.57		Lowlands	12.69	12.22		MKS	3.26	5.85	

MS: Mountain forest in Mau region; MS: Savanna land in Mau region; MKS: Savanna land in Mount Kenya.

Table S8. Three variables used in latent class analysis: COI-COII *DraI* haplotypes previously identified in [14], classical morphometrics andr7-*frag* nucleotide diversity cluster. All three data sets were obtained from the same individuals.

COI-COII DraI Haplotype			Morphometric Ecotype			r7-*frag* Nucleotide Diversity Cluster		
**Level**	**N**	**%**	**Level**	**N**	**%**	**Level**	**N**	**%**
A1	2	3.2	Highland	20	30.3	1	12	18.2
O5'	1	1.6	Lowland	26	39.4	2	12	18.2
Y1	8	12.9	Midland	20	30.3	3	42	63.6
Y2	39	62.9						
Y3	12	19.4						
Total	62	100		66	100		66	100

Table S9. Summary of samples with sequence gap (denoted as *d*) at position 858to 915 of the nuclear marker r7-*frag* on chromosome seven in the honey bee.

Country	Local Area	AEZ	N	Proportion of Sample with d		Proportion of Sample without d	
				n	%	n	%
Ethiopia	Mugulat	Highland	8	3	38	5	63
		Midland	9	2	22	7	78
		Lowland	7	1	14	6	86
	Werie	Highland	7	5	71	2	29
		Midland	6	1	17	5	83
		Lowland	11	2	18	9	82
	Koyetsa	Highland	9	3	33	6	67
		Midland	11	3	27	8	73
		Lowland	9	0	0	9	100
	Wendogenet	Midland	7	2	29	5	71
		Lowland	10	0	0	10	100
			94	22	23	72	77
Kenya	Mount Mau	Forest (MF)	10	2	20	8	80
		Savannah (MS)	17	10	59	7	41
	Mount Kenya	Savannah (MKS)	18	5	28	13	72
	All savannah		35	15	43	20	57

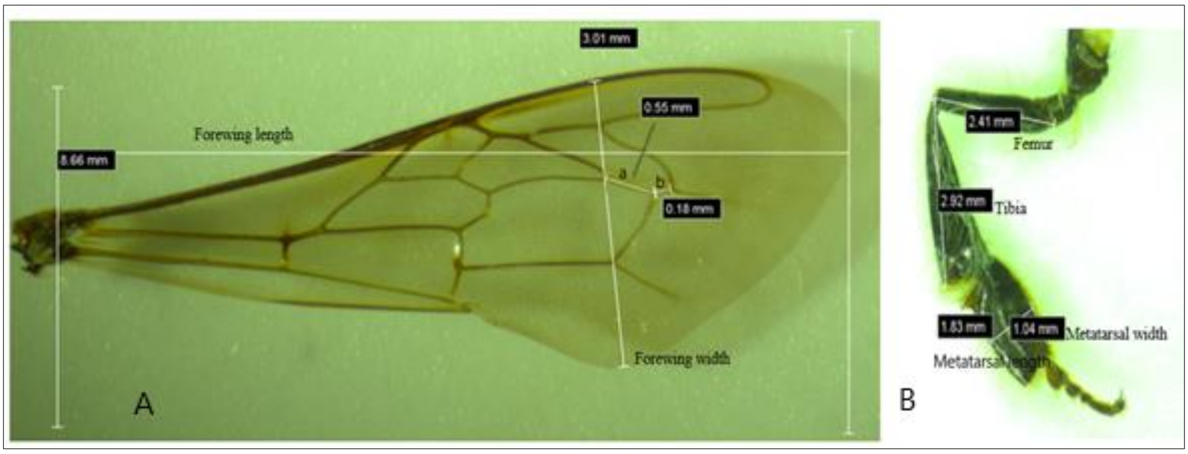

Figure S1. Selected morphometric traits out of forewing (**A**) and hind leg (**B**) of the sample honey bee analyzed in this study: femur, tibia, metatarsus length and width, metatarsal index, hind leg length, forewing length and width, cubital vein distances a and b, and cubital index.

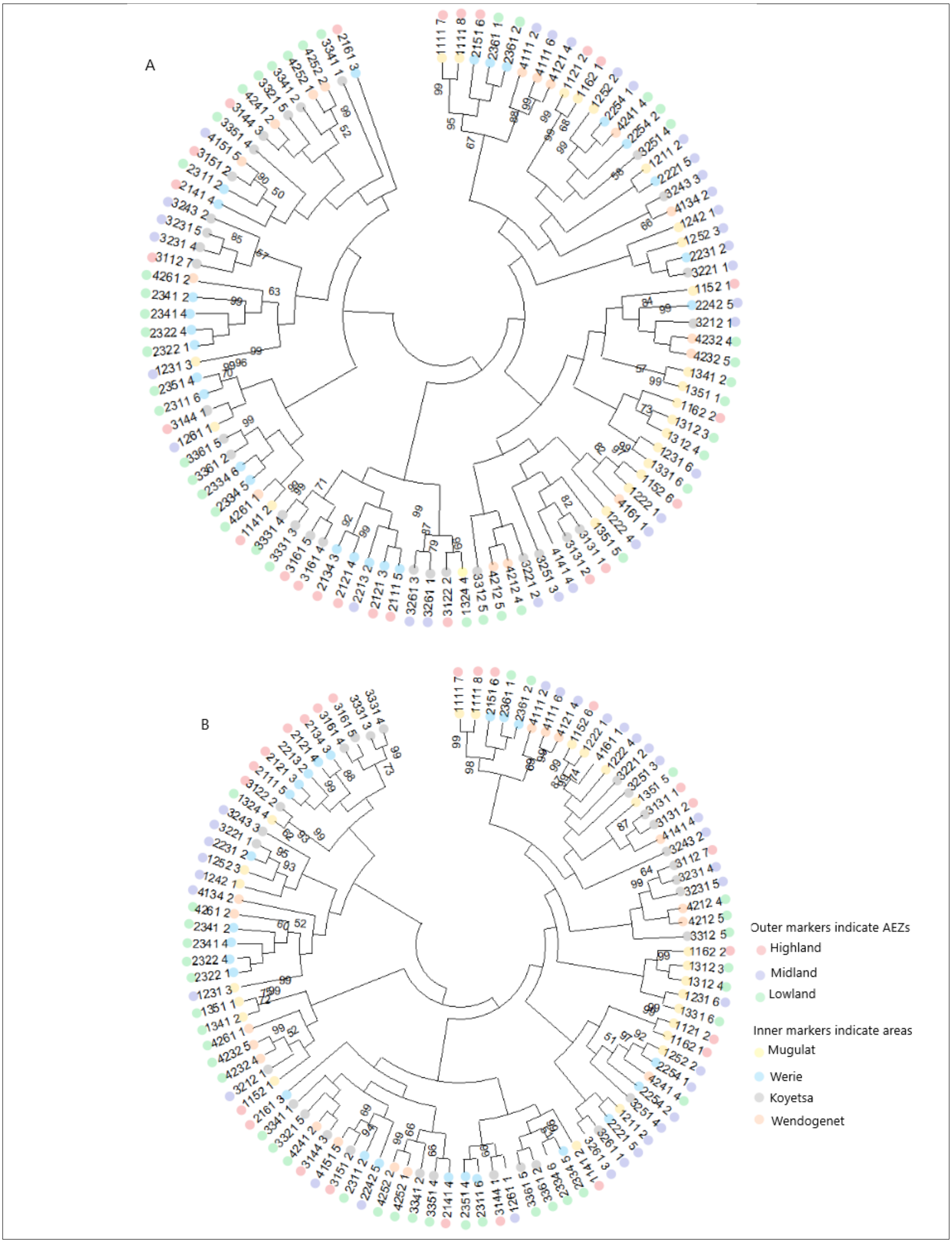

Figure S2. Phylogenetic trees of this study sequences out of three agroecological zones (AEZs) in four local areas: The evolutionary history was inferred by using Maximum Likelihood method and Tamura-Nei model [43]. Initial trees for the heuristic search were obtained automatically by applying Neighbor-Join and BioNJ algorithms to a matrix of pairwise distances estimated using the Maximum Composite Likelihood (MCL) approach, and then selecting the topology with superior log likelihood value. A discrete Gamma distribution was used to model evolutionary rate differences among sites (5 categories (+G, parameter = 0.4080)). The rate variation model allowed for some sites to be evolutionarily invariable ([+I], 37.83% sites). This analysis involved 94 nucleotide sequences. (A) All positions containing gaps and missing data

were eliminated and there were a total of 1644 positions in the final dataset. (B) Using all sites with a total of 1996 positions in the final dataset. Evolutionary analyses were conducted in MEGA X [42].

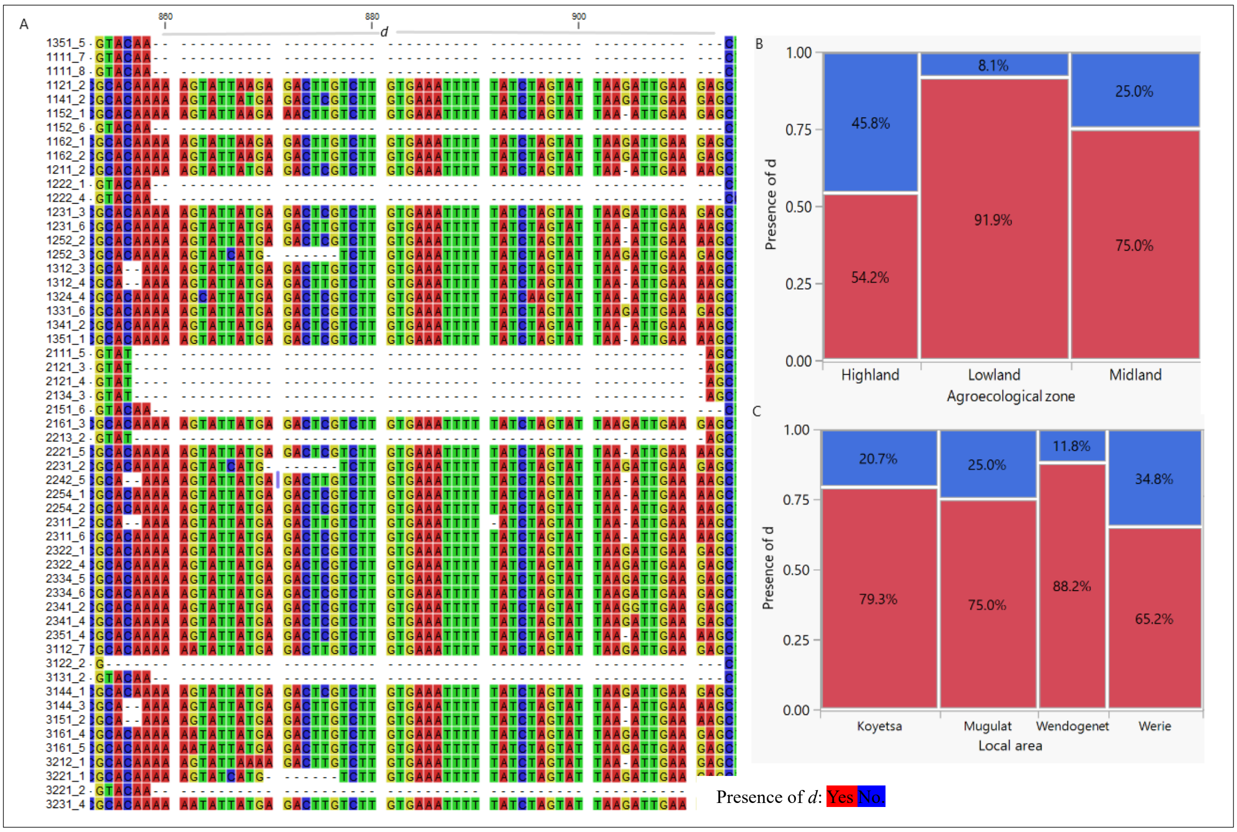

Figure S3. Demonstration and analysis of an allelic length polymorphism containing a sequence gap of 55 nucleotides denoted as d. (**A**) A partial view of this study sample sequences indicated with IDs on the left margin showing position 858 to 915 of the putative candidate r7-*frag* on chromosome seven in the honey bee. (**B**) Contingency analysis of d distribution by agroecological zones (AEZs), which strongly associated ($X^2 = 11.84$, $p < 0.01$). (**C**) Contingency analysis of d distribution in three local areas (Mugulat, Werie, Koyetsa) of northern Ethiopia consisting of highland, midland, lowland and Wendogenet area in southern Ethiopia consisting of midland and lowland AEZs.

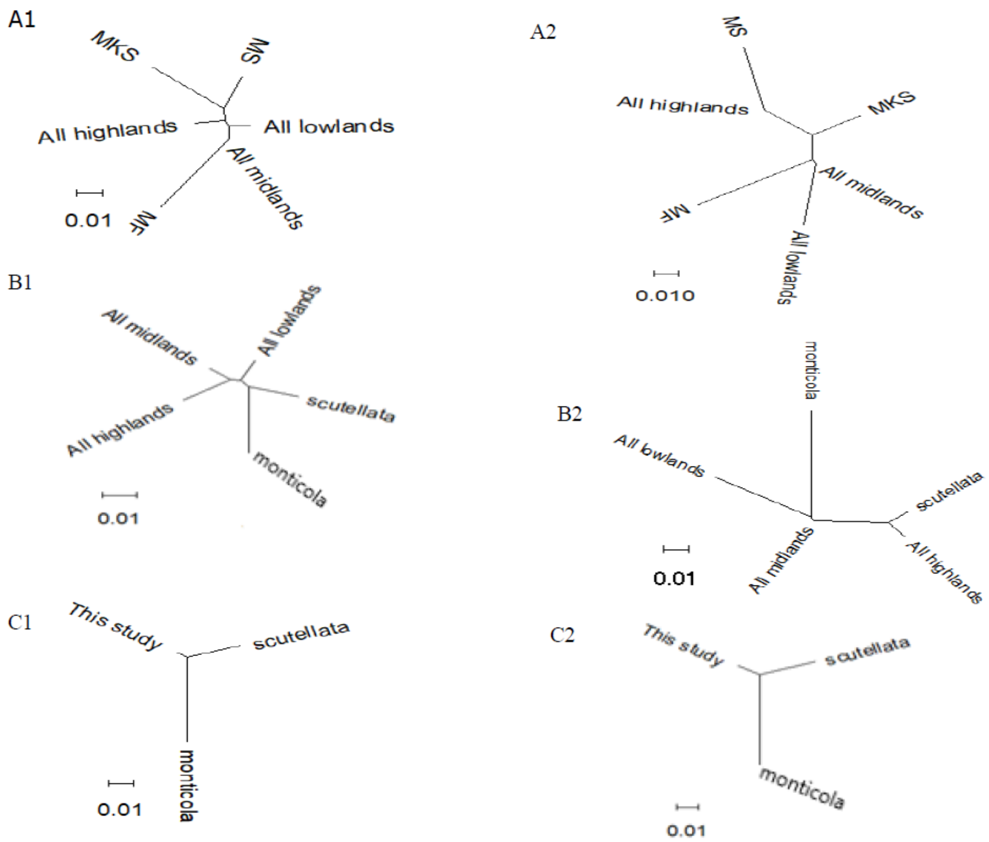

Figure S4. Neighbor-Joining trees based on pairwise distances (F_{ST}) using r7-*frag* sequence information obtained from honey bees from different agro-ecological zones (AEZs) in Mugulat, Werie, Koyetsa and Wendogenet areas of Ethiopia and *A. m. monticola* and *A. m. scutellata* samples from Kenya. Samples per AEZ (highland, midland, lowland) were pooled. (1) F_{ST} analysis conducted by excluding sites with sequence alignment gaps. (2) F_{ST} analysis conducted by including gap as a fifth state amplifying distances. (**A**) Differentiation between honey bee populations from highland, midland and lowland AEZs in reference to samples from Mau forest area (MF) and savannah area of Mount Kenya (MKS) and Mau region (MS) depicting divergence of Ethiopian highland bees away from Kenyan mountain forest bees (**B**) Differentiation between honey bee populations from highland, midland and lowland AEZs in reference to *A. m. monticola* and *A. m. scutellata* samples from Kenya indicating that scutellata is relatively closer to the honey bees in all AEZs of Ethiopia (**C**) Divergence of this study samples of Ethiopia relative to Kenyan reference monticola and scutellata. B and C indicate a marked divergence of *A. m. monticola* from both *A. m. scutellata* and Ethiopian samples of this study. Evolutionary analysis was performed using MEGA X [48] and genetic differentiation with DnaSP 6.12.03 software [41]. Note: all highlands: samples from the highland AEZs of Mugulat, Werie and Koyetsa; all midlands: samples from the midland AEZs of Mugulat, Werie, Koyetsa and Wendogenet; all lowlands: samples from the highland AEZs of Mugulat, Werie, Koyetsa and Wendogenet; this study: samples of this study collected from Ethiopia.

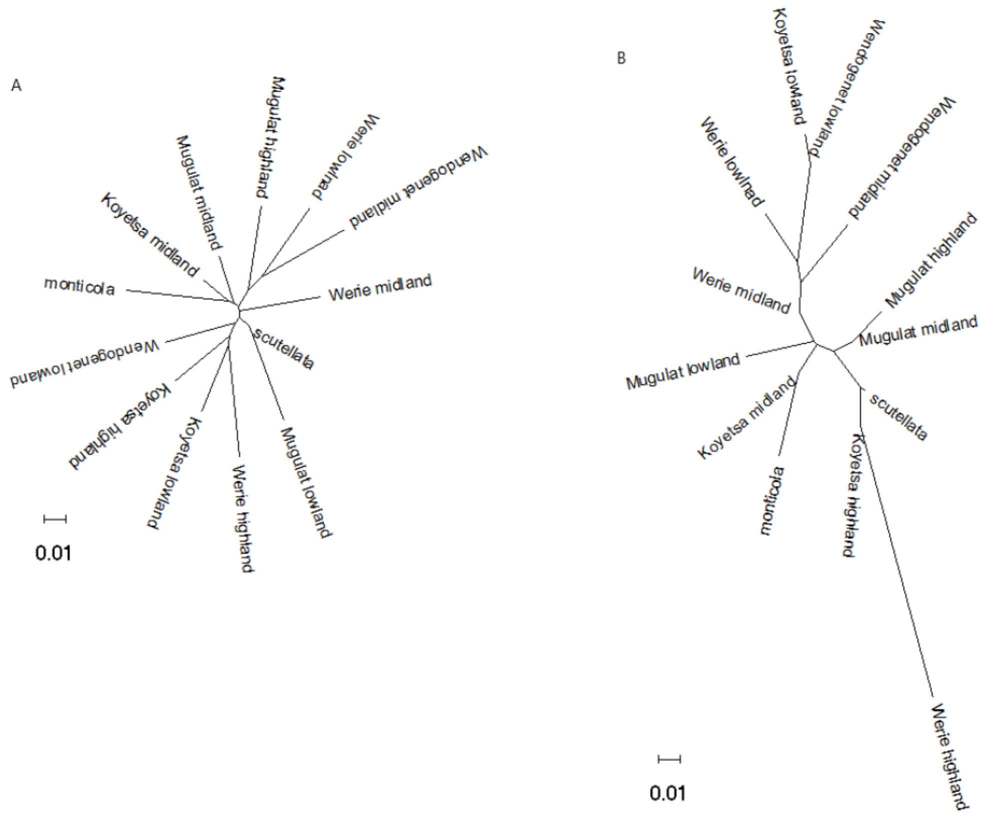

Figure S5. Neighbor-Joining (NJ) trees based on pairwise distance (F_{ST}) using r7-*frag* sequence information obtained from honey bees in highland, midland, lowland agro-ecological zones (AEZs) in Mugulat, Werie, Koyetsa and Wendogenet areas of Ethiopia and *A. m. monticola* and *A. m. scutellata* samples from Kenya. (**A**) NJ tree based on F_{ST} values analyzed by excluding sites with sequence alignment gaps. (**B**) NJ tree based on F_{ST} values analyzed by including gap as a fifth state showing marked differentiation between honey bees of lowland and highland AEZs in Werie due to a large segment of allelic length polymorphism mainly position 858 to 915 of r7-*frag* where a sequence gap of 55bp denoted as *d* characterized the highland in contrast to the lowland bees in these areas. Evolutionary analysis was performed using MEGA X [48] whereas genetic differentiation was conducted in DnaSP 6.12.03 software [41].
